# Electricity from lignocellulosic substrates by thermophilic *Geobacillus* species

**DOI:** 10.1038/s41598-020-72866-y

**Published:** 2020-10-12

**Authors:** Namita Shrestha, Abhilash Kumar Tripathi, Tanvi Govil, Rajesh Kumar Sani, Meltem Urgun-Demirtas, Venkateswaran Kasthuri, Venkataramana Gadhamshetty

**Affiliations:** 1 Civil and Environmental Engineering, South Dakota School of Mines and Technology, Rapid City, SD 57701 USA; 2 Department of Civil and Environmental Engineering, Rose-Hulman Institute of Technology, Terre Haute, IN 47803 USA; 3 Department of Biological and Chemical Engineering, South Dakota School of Mines and Technology, Rapid City, SD 57701 USA; 4 BuGReMeDEE Consortium, South Dakota School of Mines and Technology, Rapid City, SD 57701 USA; 5 Energy Global Security Division, Argonne National Laboratory, Lemont, IL 60439 USA; 6 Biotechnology and Planetary Protection Group, Jet Propulsion Laboratory, California Institute of Technology, Pasadena, CA 91109 USA

**Keywords:** Biofilms, Microbiology

## Abstract

Given our vast lignocellulosic biomass reserves and the difficulty in bioprocessing them without expensive pretreatment and fuel separation steps, the conversion of lignocellulosic biomass directly into electricity would be beneficial. Here we report the previously unexplored capabilities of thermophilic *Geobacillus* sp. strain WSUCF1 to generate electricity directly from such complex substrates in microbial fuel cells. This process obviates the need for exogenous enzymes and redox mediator supplements. Cyclic voltammetry and chromatography studies revealed the electrochemical signatures of riboflavin molecules that reflect mediated electron transfer capabilities of strain WSUCF1. Proteomics and genomics analysis corroborated that WSUCF1 biofilms uses type-II NADH dehydrogenase and demethylmenaquinone methyltransferase to transfer the electrons to conducting anode via the redox active pheromone lipoproteins localized at the cell membrane.

## Introduction

Exoelectrogens are microorganisms that use a unique extracellular electron transfer (EET) capabilities to generate electricity from organic substrates in microbial fuel cells (MFCs)^1–3^. The EET mechanisms^4^ represent the earliest mode of energy conservation in thermophilic microorganisms that survived the volcanic environments of the early earth^5^. For example, a hyperthermopihilic *Thermotoga maritima spp.* use insoluble metals such as iron(III) (hydr)oxides as the terminal electron acceptors to conserve energy in the harsh environments characterized by hot and anaerobic conditions^6^. Biogenic magnetite in the ancient rocks is also a classic signature of exoelectrogenicity in thermophiles^7,8^. This exoelectrogenicity^9^ may allow certain thermophiles to generate electricity directly from lignocellulosic biomass.

Exoelectrogens use multiheme c-type cytochromes, conductive nanowires, or redox-active electron shuttles to generate electricity. Most studied exoelectrogens so far include Gram-negative bacteria (e.g., *Geobacter sulfurreducens)*^10,11^ and none of them contain enzymes required to hydrolyze complex polysaccharides. *Geobacillus* spp. are Gram-positive thermophilic aerobic or facultatively anaerobic bacteria that contain ligninolytic and cellulolytic enzymes^12–15^ and can hydrolyze complex polysaccharide components^16^ typical to lignocellulosic biomass^17,18^. Our limited knowledge on the EET mechanisms^19^ in these Gram-positive Firmicutes is hindering their use in MFCs.

Gram-positive bacteria contain unique cell wall characteristics^20^, including thicker peptidoglycan layer, lack of outer cell membrane, presence of teichoic acids and their enclosure in glycoprotein S layer^21,22^, all of which can mask their exoelectrogenic characteristics. Recent studies have begun to reveal presence of Gram-positive bacteria in exoelectrogenic biofilms^23^ and a promise for presence of the EET mechanisms in these bacteria^24–26^. Here we explore the exoelectrogenic capabilities of a facultative anaerobic *Geobacillus* sp. strain WSUCF1 that was isolated by the authors’ group from hot environments of a composting facility. In earlier studies, strain WSUCF1 was found to degrade lignocellulosic biomass under thermophilic and facultative anaerobic conditions ^27^. It secretes large amount of extracellular polymeric substance^28^ which may play a key role in the establishment and maintenance of biofilm structure^29^.

Here we explore and demonstrate the ability of strain WSUCF1 to generate electricity under thermophilic conditions from corn stover and food waste, the model substrates for lignocellulosic biomass and complex organic wastes, respectively. We used a combination of proteomics and genomics analysis, electrochemistry methods and chromatography tests to discern the EET mechanisms of WSUCF1 biofilms.

## Results and discussion

### Electricity generation from pure glucose by *Geobacillus* sp. strain WSUCF1.

**Figure 1:**
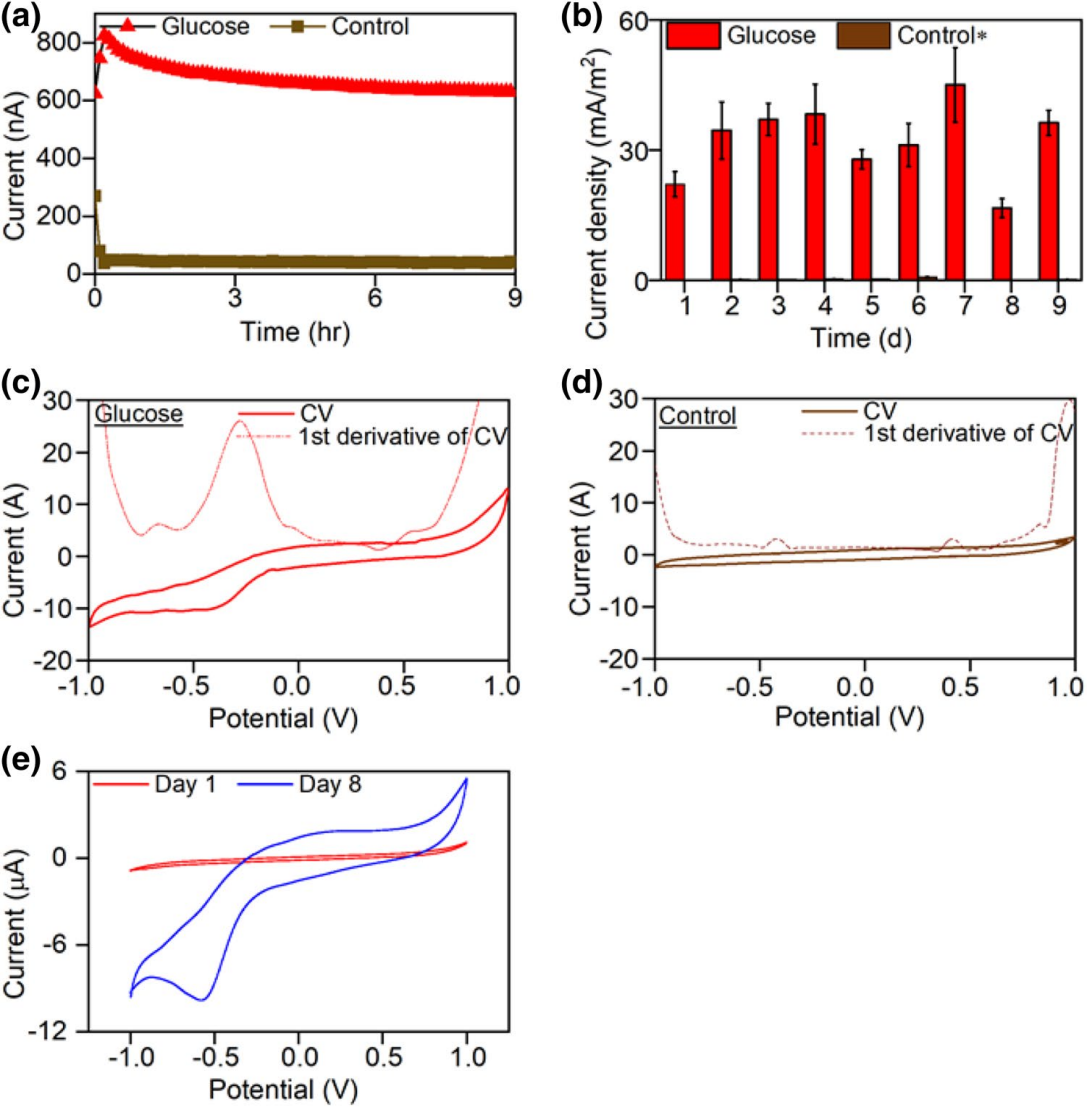
Electricity generation by Geobacillus sp. strain WSUCF1 from glucose under a cyclic fed-batch 3-EC mode. (**a**) Temporal current profiles; (**b**) Daily current density profiles; (**c**) First derivate of cyclic voltammetry for control; (**d**) First derivate of cyclic voltammetry for glucose; (**e**) Cyclic voltammetry curve for WSUCF1 in glucose substrate (Day 1 vs Day 8). Notes: All experiments were carried out in triplicates in 3-electrode electrochemical cell. (*Current range for Control: 0.02 to 0.12 mA/m^2^). See Table S1 for experimental details of 3-EC.

Upon inoculation with WSUCF1 cells in the 3-electrode electrochemical cell (3-EC), operated in a fed batch mode that entailed dual cycles, the voltage followed a gradually increasing pattern and stabilized at 0.065 µA (Fig. 1a). Operational details of the cyclic fed-batch processes including length of cycles are provided in Table S1. The peak values of the open circuit voltage at the optimum growth temperature of 60 ^O^C were 400 ± 50 mV and the current density were 45 ± 10 mW/m^2^, respectively (Fig. 1b). The poising potential of − 0.2 V (vs Ag/AgCl) was chosen based on the repeatable stable current output that we observed in the 3-EC tests (Figure S2, Supplementary Information).

The first derivative analysis of the cyclic voltammograms (CVs) at low scan rate (10 mv/s) were used to determine the peak potentials and inflection points of the glucose oxidation by WSUCF1 (Fig. 1c,d). A dominant inflection point appeared at − 0.272 V versus Ag/AgCl and a minor peak at − 0.652 vesus Ag/AgCl. These peaks appeared consistently in the CV tests with all the other scan rates (Figure S6, Supplementary Information). The control was devoid of any apparent biocatalytic current (Fig. 1d). The ability of WSUCF1 to transfer the electrons from glucose oxidation to the glassy carbon electrode was corroborated after observing a distinct current response from the cyclic voltammetry (CV) curve (8 days after the inoculation, Fig. 1e).

**Figure 2:**
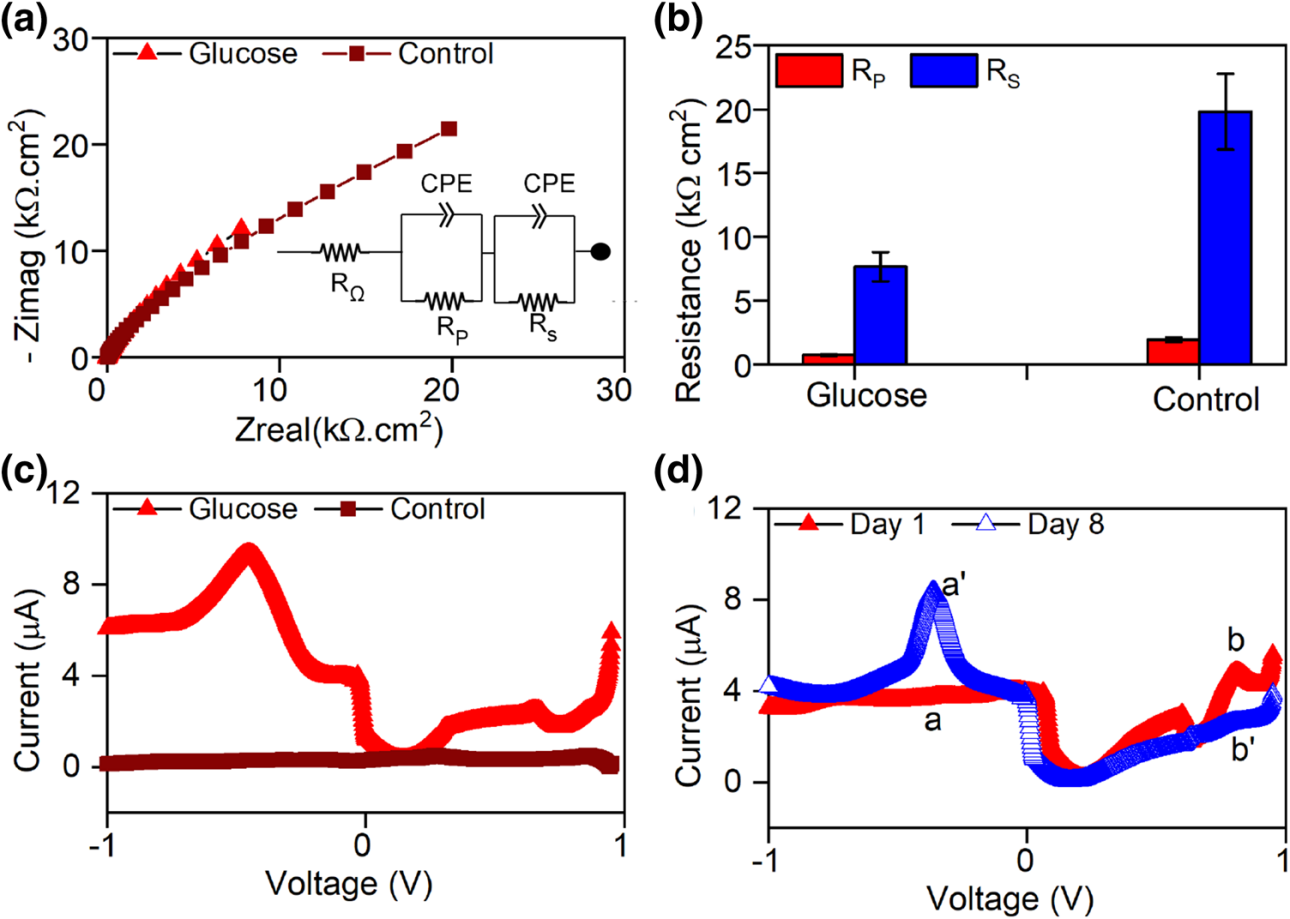
Electrochemical response of *Geobacillus* sp. strain WSUCF1 in glucose. (**a**) Nyquist plots for the entire frequency range (10 kHz to 10 MHz) (Inset shows electrical equivalent circuit used for fitting the EIS data); (**b**) Fitting results; (**c**) Representative differential pulse voltammetry scan; (**d**) DPV for glucose on temporal scale. *Note* All experiments were carried out in a 3-electrode electrochemical cell.

The Nyquist plot (Fig. 2a) and Bode plots (not shown) for WSUCF1 on Day 7 of Cycle 2 displayed two distinct time-constant regimes, one attributed to the polarization resistance (R_p_) in the high frequency region and other to substrate oxidation resistance (R_s_) in the low frequency region. The R_s_ value for the 3-EC (49.87 kΩ cm^2^) was 1.5-fold lower than that in the control (76.24 kΩ cm^2^). R_p_ (0.73 kΩ cm^2^) was also significantly lower than the control (2.8 kΩ cm^2^) (Fig. 2b). The ohmic resistance (R_Ω_) in 3-EC (0.007 kΩ cm^2^) was also 1.4-fold lower than the control (0.011 kΩ cm^2^). Among the three major sources of resistance, R_s_ was found to be the limiting resistance. The lower internal resistance in the WSUCF1 system corroborates the ability of WSUCF1 to generate electricity under thermophilic conditions.

The differential pulse voltammetry (DPV) tests corroborated the redox pairs that appeared in the CV analysis (Fig. 2c). The two DPV peaks (− 0.450 V vs Ag/AgCl and 0.67 V vs Ag/AgCl) indicated the presence of redox active center on the surface of WSUCF1 cells. These DPV results were highly reproducible as the peak was always centered at − 0.45 V vs Ag/AgCl (One-way ANOVA, *p* value = 0.8). DPV tests revealed a broader peak which increased in height with the age of the biofilm (Fig. 2d). The increased height of peak “a'” (8.1 µA) to “a” (3.6 µA) showed the increased activity of WSUCF1 biofilm. Noting that we did not maintain strict anaerobic conditions prior to the inoculation, we attribute the peaks “b” (4.4 µA) and “b'” (2.5 µA) to the presence of dissolved oxygen. Evident from the reduced height of peak “b”, the system eventually shifted to anaerobic conditions, confirming the facultative nature of WSUCF1 in MFCs (Fig. 2d).

### Versatility of *Geobacillus* sp. strain WSUCF1 in MFCs

**Figure 3:**
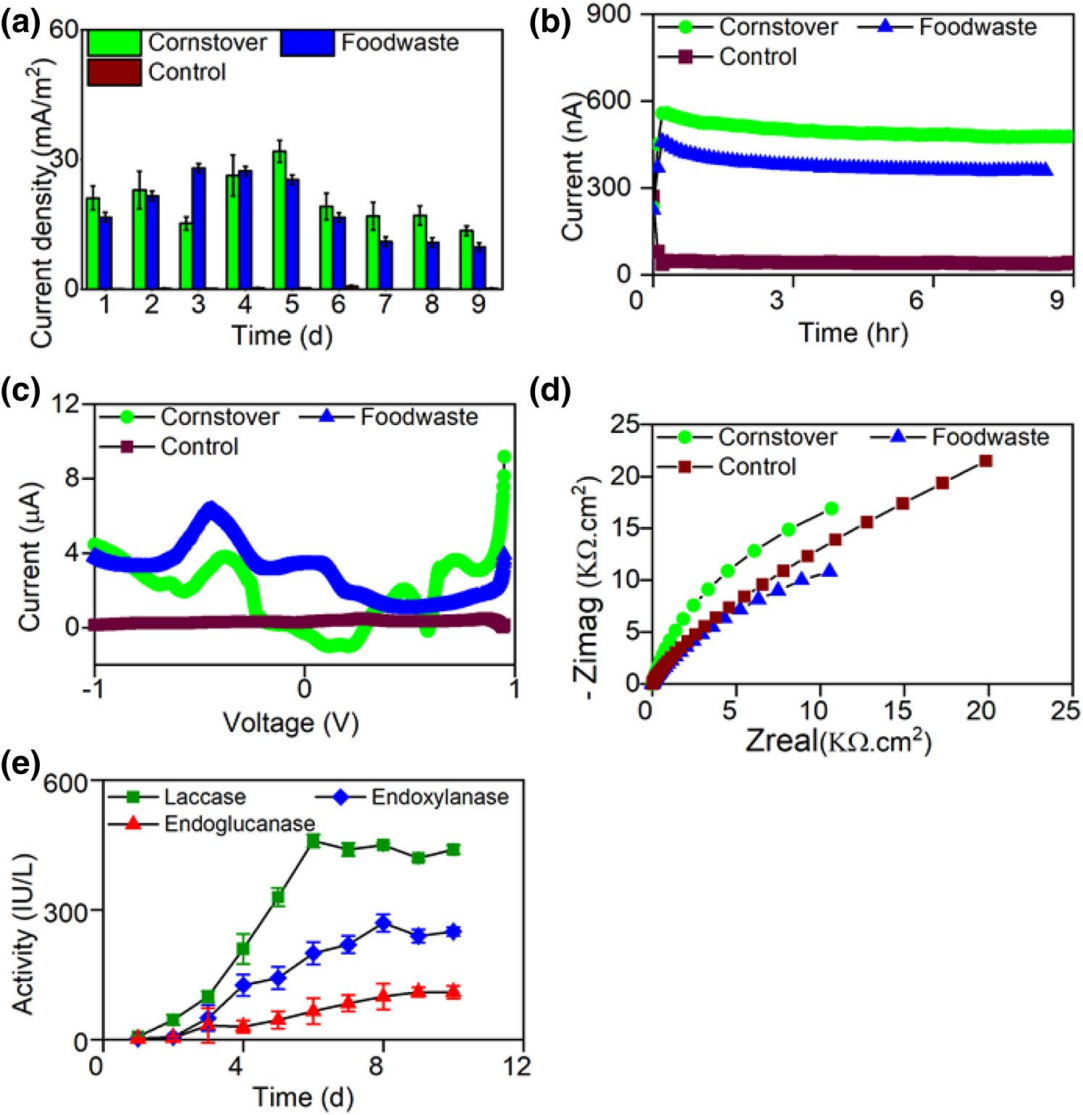
Substrate versatility analysis of strain WSUCF1 using corn stover, food waste and control (**a**) Temporal current density; (**b**) Temporal current variation; (**c**) Representative differential pulse voltammetry scan; (**d**) Nyquist plots for the entire frequency range (10 kHz to 10 MHz); (**e**) Lignolytic enzyme activities in *Geobacillus* sp. WSUCF1 in presence of 1% corn stover. Notes: All experiments were carried out in a 3-electrode electrochemical cell. Experimental details of the cyclic fed-batch processes are shown in Table S2.

The strain WSUCF1 generated electricity from both corn stover and food waste (Fig. 3a), respectively. The experimental plan for the versatility analysis of WSUCF1, based on fed-batch 3-ECs that entailed dual cycles is shown in Table S2 (Supplementary Information). Corn stover and food waste yielded peak current density of 38 mA/m^2^ and 28 mA/m^2^ in Cycle 3 of the fed-batch operation, respectively. Although these values are lower than glucose (45 mA/m^2^) (Figs. 1a, 3a), their current density profiles were devoid of any undesirable lag phase and they both yielded consistent current profiles (Fig. 3b). The CV peaks for corn stover and food waste were centered at − 0.368 V vs Ag/AgCl and − 0.442 V vs Ag/AgCl, respectively (Fig. 3c). Their electrochemical impedance spectroscopy (EIS) profiles were equivalent to that of pure glucose substrate (Nyquist plots, Fig. 3d), which confirmed the ability of the strain WSUCF1 to reduce charge transfer resistance to the Faradaic reactions influencing the oxidation of complex substrates. The three EIS profiles were carried under identical experimental conditions and on Day 5, Cycle 3 of the fed-batch operation.

To examine the reasons that allowed WSUCF1 to treat unprocessed corn stover in absence of any physico-chemical pretreatment, we assessed the presence of endogenous hydrolyzing enzymes in WSUCF1 cultures. The methods used for measuring the enzyme activities are given in the supplementary information. The enzyme assay studies revealed cellulase, hemicellulases and laccase in the WSUCF1 cultures (Fig. 3e). The 10-day growth profile indicated highest activity of laccase in supernatant on day six (460 IU/L), endoxylanase on day eight (270 IU/L), and endoglucanase on day ten (110 IU/L). A data mining exercise revealed the presence of genes encoding for hemicellulases (xylan 1,4-β-xylosidase, endo-1,4-β-xylanase, and xylan α-1,2-glucuronosidase), cellulases (endoglucanases, -glucosidases) and amylases in the genome of strain WSUCF1^28,30–32^. We observed that these enzymes displayed stable activity even after 10 days of the prolonged exposure at 60 ^O^C, corroborating the thermostability of these enzymes, as reported in previous studies ^33,34^ (Fig. 3e).

### Mechanisms of extracellular electron transfer in *Geobacillus* sp. strain WSUCF1

**Figure 4:**
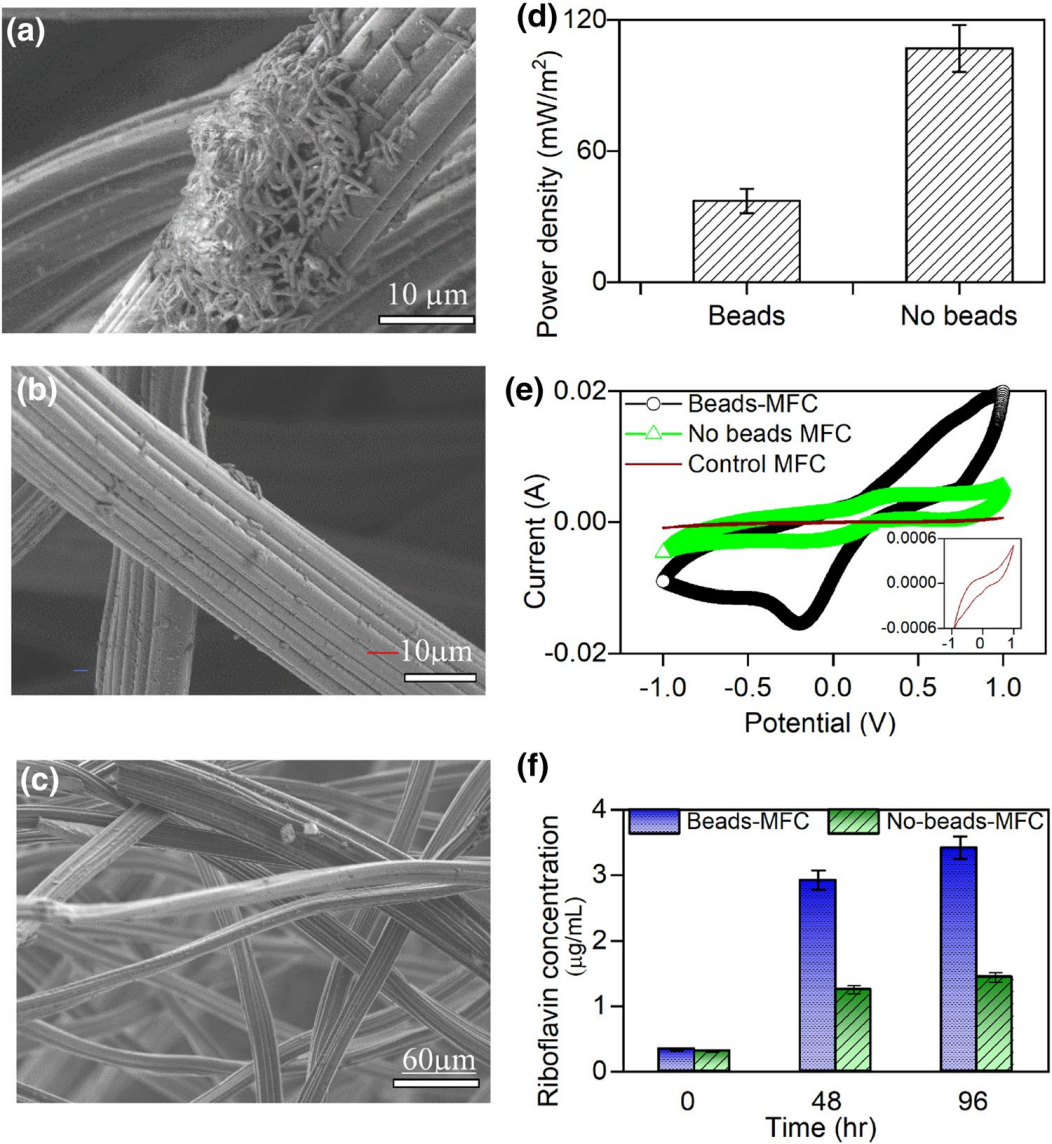
Microbial fuel cell beads/no beads experiment scanning electron microscopy images. (**a**) No-beads-MFC; (**b**) Beads-MFC; (**c**) Control-MFC; (**d**) Representative Power density; (**e**) Cyclic voltammetry (Inset shows voltammogram shape of control-MFC); (**f**) HPLC data showing riboflavin concentration.

Our final goal is to elucidate the mechanism of extracellular electron transfer (EET) in WSUCF1. We carried out “Beads /No beads” tests, using cyclic fed-batch strategies shown in Table S3 (Supplementary Information) to ascertain the roles of mediated electron transfer (MET) mechanisms in WSUCF1. To avoid the growth of WSUCF1 biofilm on the anode surface in beads-MFC, the WSUCF1 cells were encapsulated in the calcium alginate beads prior to introducing them into the MFCs. Owing to the nano-scale dimensions of typical beads [(Φ = 6.8 to16.6 nm)^11^, extremely small inter-bead distance and high specific surface area (2.486 cm^2^g^−1^)], we expect the beads to fully encapsulate the micron-scale WSUCF1 cells (Φ = 1 µm)^15–18^ and prevent them from leaking into the anolyte. Furthermore, the calcium alginate beads remain stable at the operating temperatures considered in this study (60 °C) ^19^. Thus the electricity generation in beads-MFC was primarily due to the planktonic cells. As shown, the WSUCF1 cells in no-beads-MFC adhered readily on the anode surface (Fig. 4a) compared to beads-MFC (Fig. 4b) and control (Fig. 4c).

The power density (107 mW/m^2^) in no beads-MFC was 2.9-fold higher compared to beads-MFC (37 mW/m^2^) (Fig. 4d). The performance of no-beads-MFC stayed intact even after the media replacement (42 and 46 mW/m^2^) before and after the media replacement respectively; Fig. 4a, b, S7), whereas beads-MFC experienced an outright reduction in the power output (e.g., 14 to 0.16 mW/m^2^) (data not shown). All of these results suggest the presence of endogenous redox mediators in WSUCF1-analyte. To study the nature of these mediators, CV tests using the spent-anolyte from no-beads MFC were carried out in a 3-EC cell (Details in Supplementary information). We observed distinct CV peaks whose redox potential matched with riboflavin^2^ (− 0.255 V vs Ag/AgCl) (Fig. 4e), which was further confirmed using HPLC tests (Supplementary Information). Riboflavin concentration in beads-MFC was one–threefold higher compared to no-beads MFC (Fig. 4f). Details of the anolyte samples used for riboflavin measurements and methods of riboflavin analysis are described in Sect. 4.2 (Supplementary Information).

When the biofilm forming capability of WSUCF1 was obviated by encapsulating the cells in beads, WSUCF1 secreted redox-active riboflavin to trigger the MET process. We used proteomics and genomics analysis to study the electron carrier proteins in the cytoplasm and outer membrane responsible for the MET processes. The anolyte samples used, and methods of proteomics and genomic analysis are described in Sects. 5 and 6, respectively (Supplementary Information). Among the analyzed proteins from the cytosolic and membrane protein fractions, a total of 11,427 unique peptides corresponding to 1720 different proteins were revealed. This study revealed various known proteins including cytochrome proteins involved in EET (Table 1) and nearly 154 uncharacterized proteins (Table S5, Supplementary Information) that are likely affiliated to the intracellular electron transport chain. These proteins were screened by identifying the subcellular localization of proteins using the PSORTb v.3.0.2 tool^35^ and the screened proteins were assigned a function using the PROSITE database tool (prosite.expasy.org and predictprotein.org). PSORTb tool refers to the protein subcellular localization tool. The EET proteins identified from genomics analysis of WSUCF1 are shown in Table S6 (Supplementary Information).

**Table 1:** Proteins in WSUCF1 identified by LC–MS/MS analysis.

			Exclusive Unique peptides^a^	
Protein identified from LC–MS/MS analysis	Protein Subcellular Location	GO Annotation/Function ^41, 42^	Membrane Fraction	Cytoplasmic fraction
Menaquinone-cytochrome C reductase iron-sulfur subunit	Cytoplasmic Membrane	Oxidoreductase activity; oxidation reduction process	4	3
Menaquinol-cytochrome C reductase	Cytoplasmic Membrane/Multi-pass membrane protein	Electron transporter transferring electrons within cytochrome c oxidase complex activity	17	14
Cytochrome c oxidase subunit IVB	Cytoplasmic Membrane/ Spanning component of membrane	Fe oxidation	3	2
Cytochrome c oxidase subunit I	Cytoplasmic Membrane	dioxygen reduction and proton pumping across membrane; Heme Binding	3	2
Riboflavin biosynthesis protein RibBA	Cytoplasmic Membrane	Riboflavin formation	2	13
Riboflavin biosynthesis protein RibD	Cytoplasmic Membrane	Riboflavin biosynthesis	–	4
Riboflavin biosynthesis protein ribF	Cytoplasmic Membrane	Formation of riboflavin (vitamin B2), the precursor for the coenzymes flavin mononucleotide (FMN) and flavin adenine dinucleotide (FAD)	–	3
Cytochrome c oxidase subunit 2A	Cytoplasmic membrane/Transmembrane	Transfer of electrons from cytochrome c to oxygen	2	2
Cytochrome c oxidase assembly protein	Cytoplasmic Membrane	Regulation of cell redox homeostasis	3	2
NADH-ubiquinone oxidoreductase chain 4L	Cytoplasmic membrane	Transfer of two electrons from NADH to ubiquinone associated with proton translocation across the membrane	2	-
Cytochrome c class I	Cytoplasmic Membrane	Heme Binding; Metal ion Binding; Electron transfer activity	6	6
Succinate dehydrogenase or fumarate reductase, flavoprotein subunit	Cytoplasmic Membrane	Oxidoreductase activity	2	2
Cytochrome b6	Cytoplasmic membrane/Transmembrane	Iron ion binding; Electron transfer activity	2	2
Cytochrome D ubiquinol oxidase subunit I	Transmembrane	Catalysis of a redox reaction in which a diphenol or related substance acts as a hydrogen or electron donor and reduces a hydrogen or electron acceptor at low aeration rates	3	2
Cytochrome B5	Trans-cytoplasmic membrane	Electron transfer signaling	3	2
Demethylmenaquinone methyltransferase (DMM)	Cytoplasmic Membrane	Transfer of flavin molecules to extracellular electron acceptors	2	-
Cytochrome c oxidase assembly factor CtaG	Transmembrane	Oxidoreductase activity; Proton transmembrane transport	5	2
Type II NADH dehydrogenase	Cytoplasmic membrane	Catalyzes electron exchange from cytosolic NADH to lipid soluble quinone derivative ^36^	–	4
Cytochrome quinol oxidase subunit III	Cytoplasmic membrane	Proton transmembrane transport ^43^	3	3
C-type cytochrome biogenesis protein CcsB	Multipass Membrane Protein	Biogenesis of c-type cytochromes	–	2
Cluster of lipoteichoic acid synthase	Transmembrane	Involved in synthesis of teichoic acid	4	2
Electron transport flavoprotein alpha/beta subunit	Trans-cytoplasmic membrane	Mediates electron transport to extracellular acceptors^36^	10	
Phermone Lipoprotein (plp)	Cell membrane	FMN transferase activity	2	-

^a^Only those proteins were present which had an exclusive unique peptide count of 2 or more. Proteins present had 100% matching at a peptide threshold of 95% and protein threshold of 95%. Exclusive unique peptide is the number of peptide sequences exclusively unique to a protein group.

**Figure 5:**
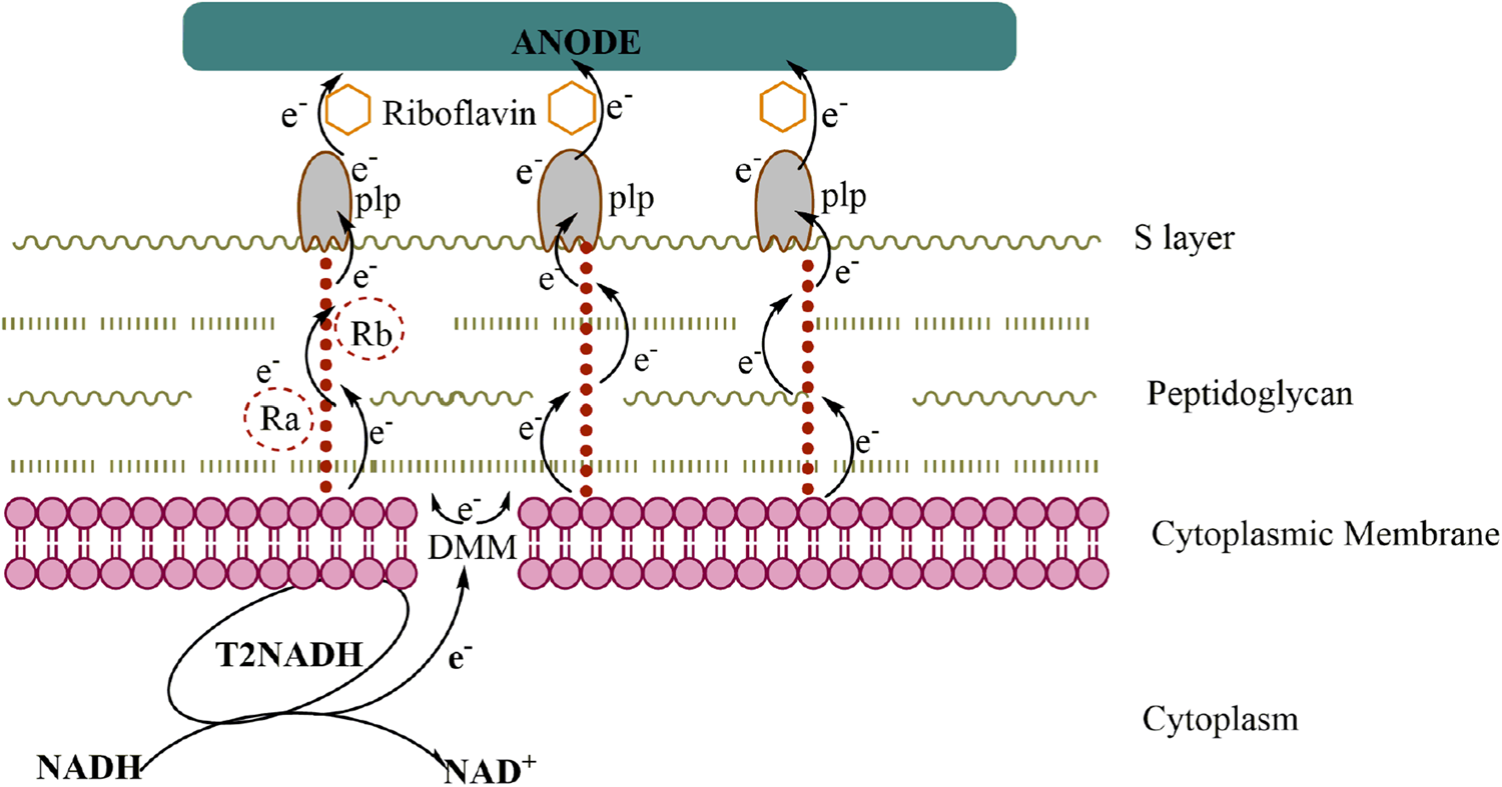
Schematic of putative Mediated electron transfer (MET) model in *Geobacillus* sp. strain WSUCF1. T2NADH, Type II NADH Dehydrogenase; DMM, Dimethyl Menaquinone Pathway; Ra and Rb, Putative redox active protein embedded in teichoic acids; plp, Outer membrane redox active phermone lipoprotein.

The proteomics analysis revealed three key proteins including type II NADH (T2NADH) dehydrogenase, demethylmenaquinone methyltransferase (DMM), pheromone lipoprotein (plp), and an anionic glycopolymer (teichoic acid). Here we establish the putative MET pathways in strain WSUCF1 based on the four key proteins (Fig. 5). The electrons from the glucose oxidation enter the electron transport chain through NADH ubiquinone oxidoreductase, ubiquinone pool, and transmembrane cytochrome proteins. The EET is initially aided by the T2NADH dehydrogenase^36^ because T2NADH diverts the intracellular electrons away from aerobic respiration and funnel them into the quinone pool localized within the cytoplasmic membrane (Table 1). Quinones such as demethylmenaquinone, menaquinone and ubiquinone then selectively funnels the electrons into riboflavin^37^. We attribute the DMM protein (Table 1) to play a key role in facilitating the MET by riboflavin. The electrons from DMM to plp on the cell surface occur via some putative redox active proteins and cytochromes (R_a_ and R_b_) positioned along the teichoic acids anchored to the peptidoglycan layer. The mining of of WSUCF1 genome confirmed the presence of all the four genes of riboflavin operon which were previously reported for *Bacillus subtilis*^38,39^*,* a native riboflavin producer (Table S4, Supplementary Information). The presence of plp (Table 1) annotated as having FMN transferase activity transfers the electrons through riboflavin to the graphite electrode^36,40^.

In summary, the current study established the extracellular electron transfer capabilities of a thermophilic, Gram positive *Geobacillus* sp. strain WSUCF1 which allow them to liberate the electrons from the complex carbon substrates onto conductive solid electrodes. Here a Gram positive *Geobacillus* sp. strain WSUCF1 generated electricity directly from corn stover using its unique pathways of lignocellulolytic reaction, biofilm formation, and transmembrane and extracellular electron transfer pathways. Altogether thermophilic Firmicutes from *Geobacillus* genera has been demonstrated to treat lignocellulosic substrates in bio-electrochemical systems. Although a clear evidence of electricity generation by WUSCF1 was shown, a series of gene-knock out studies are warranted to fully understand the metabolic network involved in the electron flow from complex wastes to the electrode. To discern the EET responses of WSCUCF1 cells exposed to steadystate physicochemical conditions, a series of studies based on continuous flow bioelectrochemical reactors are needed.

## Methods

Methods and any associated references are available in the “Supplementary Information” of the manuscript.

## Supplementary information

Supplementary Information.
