# Supplementary material for: Electricity from lignocellulosic substrates by thermophilic Geobacillus species
Source: Sci Rep. 2020 Oct 12;10:17047. doi: 10.1038/s41598-020-72866-y (PMC7552438; doi:10.1038/s41598-020-72866-y)
Supplement: Supplementary file 1 — Supplementary Information. [file 41598_2020_72866_MOESM1_ESM.docx]

**SUPPLEMENTARY INFORMATION**

**Electricity from lignocellulosic substrates by thermophilic *Geobacillus* species**

Namita Shrestha^1,2*‡^, Abhilash Kumar Tripathi^3*^, Tanvi Govil^3^, Rajesh Sani^3,4‡^, Meltem Urgun-Demirtas^5^, Venkateswaran Kasthuri^6^, Venkataramana Gadhamshetty ^1,4 ‡^

^1^Civil and Environmental Engineering, South Dakota School of Mines and Technology, Rapid City, SD 57701, USA

^2^Department of Civil and Environmental Engineering, Rose-Hulman Institute of Technology, Terre Haute, IN 47803, USA

^3^Department of Biological and Chemical Engineering, South Dakota School of Mines & Technology, Rapid City, SD 57701, USA

^4^BuGReMeDEE consortium, South Dakota School of Mines & Technology, Rapid City, SD 57701, USA

^5^Energy Global Security Division, Argonne National Laboratory, Lemont, IL 60439, USA

^6^Biotechnology and Planetary Protection Group, Jet Propulsion Laboratory, California Institute of Technology, Pasadena, CA 91109, USA.

‡ **Correspondence**: [Venkata.Gadhamshetty@sdsmt.edu](mailto:Venkata.Gadhamshetty@sdsmt.edu); [shrestha@rose-hulman.edu](mailto:shrestha@rose-hulman.edu);

**Methods**

# 1. *Geobacillus* sp. WSUCF1 Culture

A stock culture of *Geobacillus* sp. WSUCF1 was obtained from our culture collection. The growth procedures for WSUCF1 has been discussed in detail in our earlier studies^1^. Here we provide a brief overview. Axenic cultures of WSUCF1 cells from the exponential growth phase (absorbance_600nm_ = 0.4) were used as inoculum in all of the tests described herein. The optimal growth conditions for WSUCF1 cells were found to be at T = 60°C, pH = 7.0. WSUCF1 cells were grown in a defined minimal media supplemented with the specified carbon substrate (glucose, corn stover or food wastes) as described in the latter sections. The defined media consisted following per liter: 0.1 g nitrilotriacetic acid, 0.05 g CaCl_2_⋅2H_2_O, 0.1 g MgSO_4_⋅7H_2_O, 0.01 g NaCl, 0.01 g KCl, 0.3 g NH4Cl, 0.005 g methionine, 0.2 g yeast extract, 0.01 g casamino acid, 1.8 g of 85% H_3_PO_4_, 1 mL FeCl_3_ solution (0.03%), and 1 mL of Nitsch’s trace solution.

# 2. Two types of electrochemical reactors

Figure S1. A photograph of 3- EC

The experiments were carried out either in a three electrode electrochemical cell (3-EC) (Figure S1) or two-compartment MFC (Figure S3). 3-ECs were used in experiments pertinent to Figure 1, Figure 2 (pure glucose substrate) and Figure 3 (versatility analysis) and MFCs in those of Figure 4 (beads/no beads study).

3-EC Reactor: The 3-EC consists of a glassy carbon working electrode (WE), platinum counter electrode (CE) and Ag/AgCl reference electrode (RE) (Figure S1) (Pine Research Instrumentation, NC). The electrolyte was initially based on 100 mL of the defined media supplemented with 80 mL of the WSUCF1 stock solution. The inocula was introduced only at the beginning of the tests. The electrolyte was maintained at 60 ^o^C using a hot water jacketed system (the blue hoses). The WE was poised at -0.2V vs Ag/AgCl. This potential was selected as it yielded a stable current output and reproducible performance, as determined in our preliminary studies (Figure S2).

Figure S2 | WSUCF1 poised a) at 0.6 V vs Ag/AgCl b) at -0.2V vs Ag/AgCl

MFC reactor: A two-compartment MFC architecture as discussed in our earlier studies ^2-4^ was used (Figure S3). The reference electrode was Ag/AgCl electrode (Pine Research, NC) and both the anode and cathode were based on a graphite felt discs (Φ = 3 cm). The anolyte for the initial tests consisted of 13.5 mL of defined media supplemented with a specified substrate and 1.5 mL of WSUCF1 cells. (details in the latter sections). The catholyte consisted of a 15 mL of 100 mM potassium ferricyanide buffered with 100 mM phosphate buffer. The MFC along with its anolyte and catholyte were autoclaved for 20 minutes prior to introducing the inoculum. The MFCs were maintained at 60 ^o^C using a hot water bath. A DAQ/54 module (I/O Tech Inc., Cleveland OH) was used to measure the OCP, operating voltage (R_external_ = 1000 Ω) and the voltage data needed to generate the polarization curves.

Figure S3. An MFC used in the Beads-no beads study

Table S1. Electricity production by WSUCF1 from glucose in 3-EC

| **#** | **Bacteria** | **Cycle**  **(duration)** | **Tests** | **Purpose** | |
| --- | --- | --- | --- | --- | --- |
| Test  Glucose | WSUCF1 | #1 (9 d) #2 (11 d)  Total = 20 d | EIS, CV, CA, DPV | Assess feasibility of electricity production by WSUCF1; Assess electrochemical properties of WSUCF1 biofilms |  |
| Control  (No C substrate) | None | #1 (10 d)  #2 (10 d)  Total = 20 d | EIS, CV, CA, DPV | Control for WSUCF1 |  |

Note: EIS, Electrochemical impedance spectroscopy; CV, Cyclic voltammetry; CA, chronoamperometry and DPV, differential pulse voltammetry

2.1 Fed-batch mode: Both 3-ECs and MFCs were operated in a fed-batch mode. The anolyte in both MFCs and 3-ECs was based on the carbon supplemented defined media and WSUCF1. The catholyte in MFC was based on a 100 mM potassium ferricyanide solution.

The WSUCF1 cells from the exponential growth phase were used as an inoculum in Cycle I of the fed-batch operations. After achieving a steady state open circuit potential (OCV), the operation was transited into a closed circuit (CC) mode (R_ext_ = 1000 Ω). We ascertained the steady state performance under the CC mode when the operating potential (R_ext_ = 1000 Ω) remained within + 5 mV of the mean value for > 24 h. In Cycle II and beyond, our goal was to ensure that the electricity generation was solely from the pregrown WSUCF1 biofilm on the anode surface. Thus, we drained fully the spent anolyte along with its planktonic cells at end of each cycle, gently washed the anode with 50 mM phosphate, and refilled the anode compartment with an equivalent volume of fresh anolyte. Also we replaced the spent catholyte. A reproducible performance across the cycles was ascertained after ensuring that the operating potential in Cycle II was > Cycle I. We typically initiated the media replacement before the overall potential dropped < 20 mV of the average value. This strategy allowed us to avoid the substrate limiting conditions and focus on the goal of assessing WSUCF1’s ability to generate electricity from different substrates.

2.2. Electrochemical Measurements: The electrochemical performance of WSUCF1 biofilms were analyzed using a cyclic voltammetry (CV), chronoamperometry (CA), differential pulse voltammetry (DPV), electrochemical impedance spectroscopy (EIS), and linear sweep voltammetry (LSV). Reference 3000 potentiostat (Gamy Instrument Inc., PA) was used to carry out these tests. For the 3-EC, a glassy carbon electrode served as WE, platinum rod as CE and Ag/AgCl as RE. The MFC used graphite felt as WE and CE, and Ag/AgCl as RE. An AC signal (amplitude = ±10 mV) within a frequency range of 10 kHz and 10 mHz was used for all of the EIS tests.

# The EIS tests were carried out at OCP conditions. An Echem analyst software was used to fit an electrical equivalent circuit (EEC) based on the Nyquist data. A typical EEC consisted of ohmic resistance (R_Ω_), polarization resistance (R_p_) and resistance due to substrate oxidation (R_s_) (Figure 2). The DPV test parameters were E_initial_ = -0.1 V (vs Ag/AgCl), E_final_ = 1 V (vs Ag/AgCl), pulse height = 50 mV, pulse width = 300 ms, step height: = 2mV, step time = 500ms, and scan rate =1mV/s. The LSV parameters were scan rate = 1mv/s, E_initial_ = OCP, E_f_ = 0 V vs Ag/AgCl. The CV parameters included: equilibrium time = 1000s, scan rate =1mV/s; E_initial_ = 1 V vs Ag/AgCl; E_final_ =1 V vs Ag/AgCl. For CA, E_applied_= -0.2V vs Ag/AgCl was used.

# 3. Pure glucose tests and the versatility analysis study

An overview of the experiments for the glucose tests is shown in Table S1 and versatility analysis in Table S2. These tests were carried out in a 3-EC. We evaluated the performance of three test 3-ECs in two consecutive cycles extended during 20 days of fed-batch operation. The planktonic cells were removed at the end of each cycle by draining anolyte and gently washing the anode with 50 mM phosphate. For the glucose tests, the LSV data was obtained on a daily basis during the first 8 days (Cycle 1). The CV curves were generated on day 1 and day 8 (cycle One), the EIS curves on day 8 (cycle 1), and the DPV curves on day 8 (cycle 1).

For the versatility analysis studies, the LSV data was obtained on day 1 to day 8 (Cycle 1). The CV curves were generated on day 1 and day 8 (cycle 1), the EIS curves on day 8 (cycle 1), and the DPV curves on day 8 (cycle 1).

For riboflavin analysis the samples were taken every 2^nd^ day and the concentration of riboflavin was measured using the procedures described in Section 4.2. The data presented in Figure 4f is representative of the first 4 days of sampling.

For proteomics analysis, the proteins were extracted from cytosolic and membrane protein fraction of biofilm of no-beads MFC after completion of the experiment. The detailed experimental procedure of proteomics analysis is given in Section 6.

Table S2. Versatility analysis of WSUCF1

| **#** | **Carbon (g/L)** | **Bacteria** | **Cycle** | **Tests** | **Purpose** | |  |
| --- | --- | --- | --- | --- | --- | --- | --- |
| 1 | Glucose (1 g/L) | WSUCF1 | #1 (9 d) #2 (11 d)  Total (20 d) | EIS, CV, CA, DPV | Study biofilm impedance;  assess EET mechanisms | | |
| 2 | Corn stover (1 g/L) | WSUCF1 | #1 (9 d) #2 (11 d) #3 (12 d)  Total (30 d) | EIS, CV, CA, DPV | Electricity from lignocellulosic biomass  by WSUCF1 | | |
| 3 | Food waste  (1 g/L) | WSUCF1 | #1 (9 d) #2 (11 d)  Total (20 d) | EIS, CV, CA, DPV | Electricity from complex wastes  by WSUCF1 | | |
| 4 | None | None | #1 (10 d)  Total (10 d) | EIS, CV, CA, DPV | Control |  |  |

# 4. Beads/no-beads tests using two-compartment MFCs

An overview of the details for the beads/no-beads experiment is shown in Table S3. The beads/no-beads tests were carried out in two-compartment MFCs. We evaluated the performance of two test MFCs in four consecutive cycles extended during 40 days of fed-batch operation. The redox active components including FeCl_3_, nitrilotriacetic acid, casamino acid, methionine and H_3_PO_4_ were removed from the defined media to study the indigenous modes of EET in WSUCF1. Following autoclaving, 12 mL of the mineral media and 1 mL of 5 mM glucose were added in the anode compartment. Calcium alginate beads were prepared by mixing equal volumes of WSUCF1 cells (90x10^5^ CFU/mL) and sodium alginate (4% w/v) and releasing the drops of the mixture using a syringe into CaCl_2_ solution (10 wt%) ^5^. The sodium alginate beads along with the entrapped WSUCF1 cells (90x10^5^CFU/mL) were introduced into the beads-MFC. WSUCF1 cells (90 x 10^5^ CFU/mL) (lacking the entrapment approach) were injected into no-beads-MFC. An identical MFC that lacked inoculum served as a control.

Table S3. Experimental Plan MFC beads/no beads experiment

| **#** | **Cycles** | **Days/Cycle** | **Temp** | **Anode** | **Cathode** | **Electrode material** | **Culture** |
| --- | --- | --- | --- | --- | --- | --- | --- |
| Beads-MFC | 4 | 10 d/Cycle | 60 ^o^C | Media, Glucose, Trace elements | Potassium Ferricyanide | Graphite Felt | WSUCF1 entrapped in beads |
| No-beads-MFC | 4 | 10 d/Cycle | 60 ^o^C | Media, Glucose, Trace elements | Potassium Ferricyanide | Graphite Felt | WSUCF1 without beads |
| Control-MFC | 1 | 14 d/Cycle | 60 ^o^C | Media, Glucose, Trace elements | Potassium Ferricyanide | Graphite Felt | None |

# 4.1. SEM analysis for Beads/No beads test:

The biofilm-coated anode samples from day 40 were aseptically removed and examined using a Zeiss Supra 40VP field-emission scanning electron microscope (SEM) ^6^. Prior to the SEM analysis, the biofilm samples were fixed with glutaraldehyde using the fixation procedures described in our earlier study^3^.

# 4.2. Measurement and verification of Riboflavin for Beads/No beads test

Riboflavin levels in the anolyte samples were measured using the procedure described by Wang, Bai ^7^. Briefly, the samples were diluted with 0.05 M NaOH and centrifuged for 5 minutes at 16,000 g. The obtained supernatant was diluted with acetic acid sodium-acetate buffer solution (pH 5) and absorbance was recorded at 444 nm to measure riboflavin concentration ^7^. Riboflavin analysis was conducted using a Shimadzu HPLC system with a UV detector (210nm) and Shim-pack MAqC-ODS I C-18 column (150mmL. x 4.6mmI.D., 5μm). Separation was performed using the following mobile phases: (A) 10mmol/L phosphate (Na) buffer solution (pH 2.6); (B) Acetonitrile. Separation was performed at the following gradient conditions: Acetonitrile concentration 1% (0 min) → 1% (2.5 min) → 50% (10 min) → 1% (10.01 - 15 min). Flow rate of mobile phase was 1.2mL/min, and oven temperature was set at 40°C.

# 5. Enzymatic analysis

## 5.1 Preparation of crude enzymes

The WSUCF1 samples from the growth experiments were collected every 24 h. These samples were centrifuged at 8,000 rpm for 10 minutes to separate the supernatants and pellets. The supernatant was used to assess the type and quantiy of the extracellular ligninolytic enzymes. Laccase activity was determined by the oxidation of ABTS (2,2′-azino-bis (3-ethylbenzothiazoline-6-sulphonic acid)) (Sigma-Aldrich, St. Louis, MO, U.S.A.) buffered with 0.1M sodium phosphate at pH 7.0. The reaction mixture (1 mL) contained equal volumes of enzyme extract and 1 mM ABTS prepared in 0.1 M sodium phosphate buffer (pH 7). The reaction mixture was incubated at 60 °C for 10 min, and absorbance was read at 420 nm in a spectrophotometer against a suitable blank. One IU (International Unit) of laccase activity was defined as the amount of the laccase that oxidized 1μmol of ABTS substrate per min under given assay conditions. The enzyme activity was expressed in U/mL.

For endoxylanase enzyme assay, the reaction mixtures contained 1.8 mL of 1% (w/v) birchwood xylan (Sigma-Aldrich, St. Louis, MO, USA) in phosphate buffer (100 mM, pH 7.0) and 0.2 mL of the enzyme supernatant. The enzyme-substrate reaction was carried out at 60 °C for 10 min. The reaction was stopped by the addition of 3.0 mL 3,5-Dinitrosalicylic acid (DNSA) solution, boiled for 10 min, and then cooled on ice for color stabilization. The optical absorbance was measured at 540 nm, and the amounts of liberated reducing sugar (xylose equivalents) was estimated against the standard curves for xylose. One unit of xylanase enzyme was defined as the amount of enzyme that releases 1 µmol of xylose per minute under reaction conditions.

For cellulase enzyme assay, the reaction mixtures contained 1.8 mL of 2% (w/v) CMC- carboxymethyl cellulose (Sigma-Aldrich, St. Louis, MO, USA) in phosphate buffer (100 mM, pH 7.0) and 0.2 mL of the enzyme supernatant. The enzyme-substrate reaction was carried out at 60 °C for 10 min. The reaction was stopped by the addition of 3.0 mL 3,5-Dinitrosalicylic acid (DNSA) solution, boiled for 10 min, and then cooled on ice for color stabilization. The optical absorbance was measured at 540 nm, and the amounts of liberated reducing sugar (xylose equivalents) was estimated against the standard curves for glucose. One unit of xylanase enzyme was defined as the amount of enzyme that releases 1µmol of glucose per minute under reaction conditions.

# 6. Proteomic Analysis

The WSUCF1 cells in the biofilm on the graphite anode samples were obtained on day 10 (cycle 4) of the no-beads MFC tests. The biofilm was harvested from electrode surface by scraping and using a sonication process in 100mM phosphate buffer saline as described previously ^8^. A 50 mL aliquots of the scrapped cells was pelleted by centrifuging them at 10,000 g for 5min at 4°C.

## 6.1 Extraction of cytosolic and membrane proteins

The preparation of membrane protein from the harvested biofilm cultures was performed as follows. The cell pellet was washed thrice with 100 mM phosphate-buffered saline (PBS) (pH = 7.4) and washed pellet was used to prepare the membrane protein fraction. The membrane proteins were extracted using the ReadyPrep Protein Extraction Kit (membrane I) (Bio-Rad Co., Hercules, CA). The extraction process followed the steps as described in manual provided by the manufacturer. The purified membrane and cytosolic protein fractions were stored at -80°C for later analysis.

## 6.2 1-D PAGE

The membrane and cytosolic protein fractions were dissolved in 50 µL of 2X SDS sample buffer (4X Tris-HCl, pH 6.8, 4%SDS, 20% glycerol, 0.001%w/v bromophenol blue) containing 2mL of 0.2% 2-mercaptoethanol, and heated for 5 min at 100°C. The samples containing 120 µg of proteins were separated using 10% SDS-PAGE (Figure S4) and the gel was stained using pierce silver stain (ThermoFisher Scientific, Grand Island, NY).


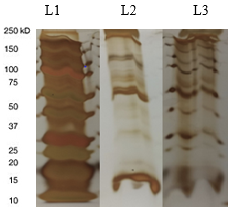


Figure S4 | 1D-PAGE of enriched membrane and cytosolic protein samples. L1: protein ladder; L2: Cytoplasmic protein fraction; L3: Membrane protein fraction. Molecular protein markers are indicated on the left (kDa)

## 6.3 LC-MS/MS analysis

The membrane and cytosolic protein fractions extract were digested by trypsin (in-solution digestion) into peptides for LC-MS/MS analysis as described previously by Gundry, White ^9^. Trypsin digested proteins were analyzed using a Q-Exactive mass spectrometer with a proxeon nano spray source connected in line with EASY-LC II HPLC. Figure S5 shows the MS/MS spectra of both membrane and cytosolic proteins. The resulting data were checked against all *Geobacillus* species database using the search engine X! Tandem, and data was analyzed using scaffold proteomics software (Thermo Scientific, Grand Island, NY). A 1% false discovery rate (FDR) was set for protein identification and peptide spectral match. Proteins with atleast 2 unique peptides were predicted to be present.

Figure S5 | MS/MS spectra for (A) Membrane proteins and, (B) Cytosolic proteins

Figure S6 | CVs for the control 3-EC and that fed with glucose at different scan rates


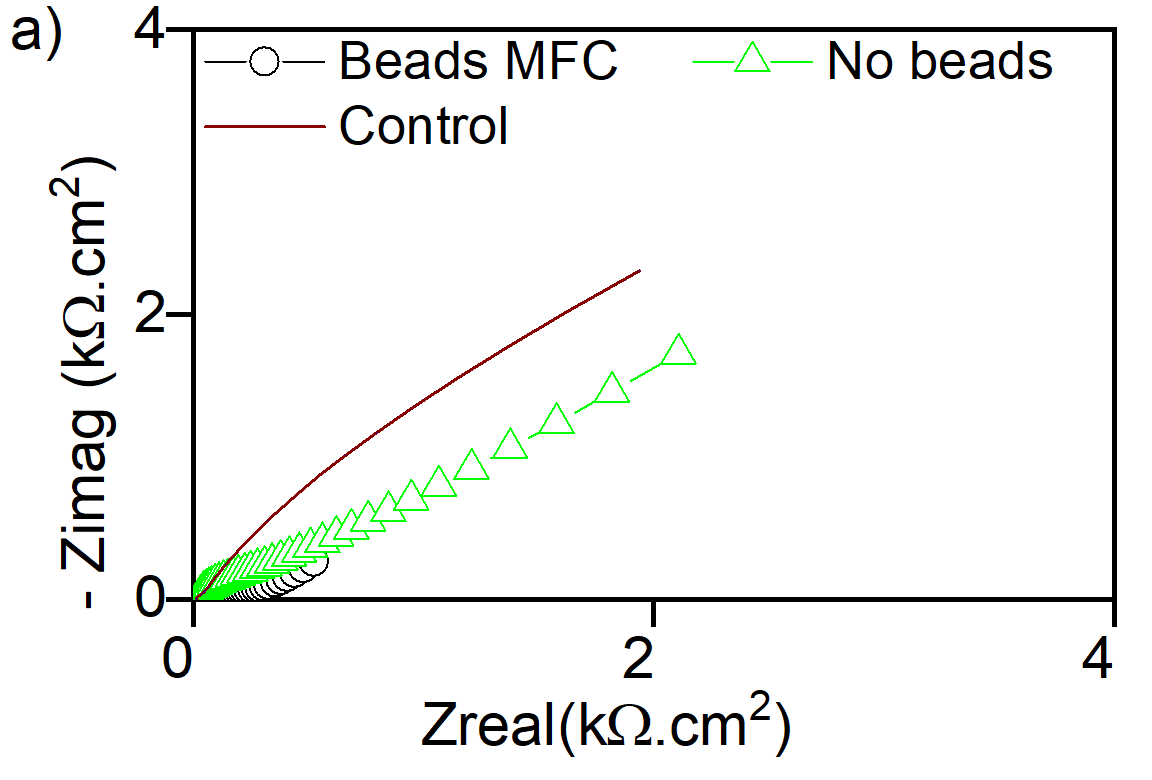

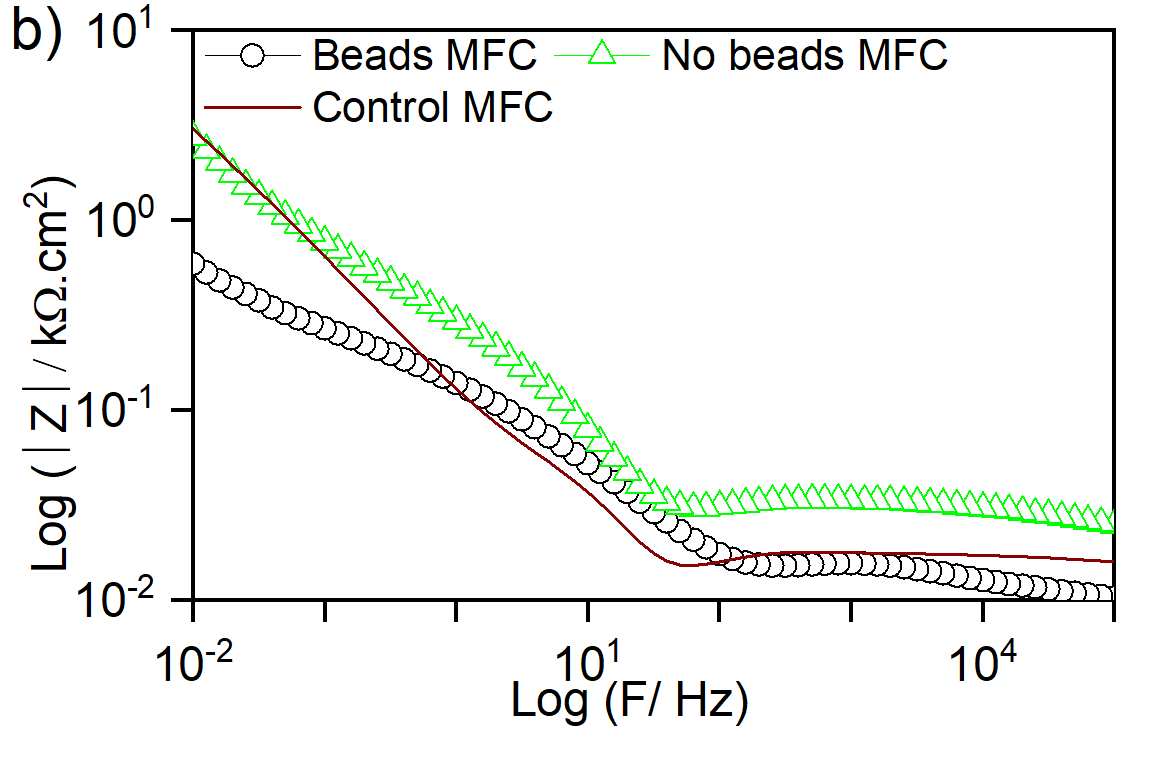

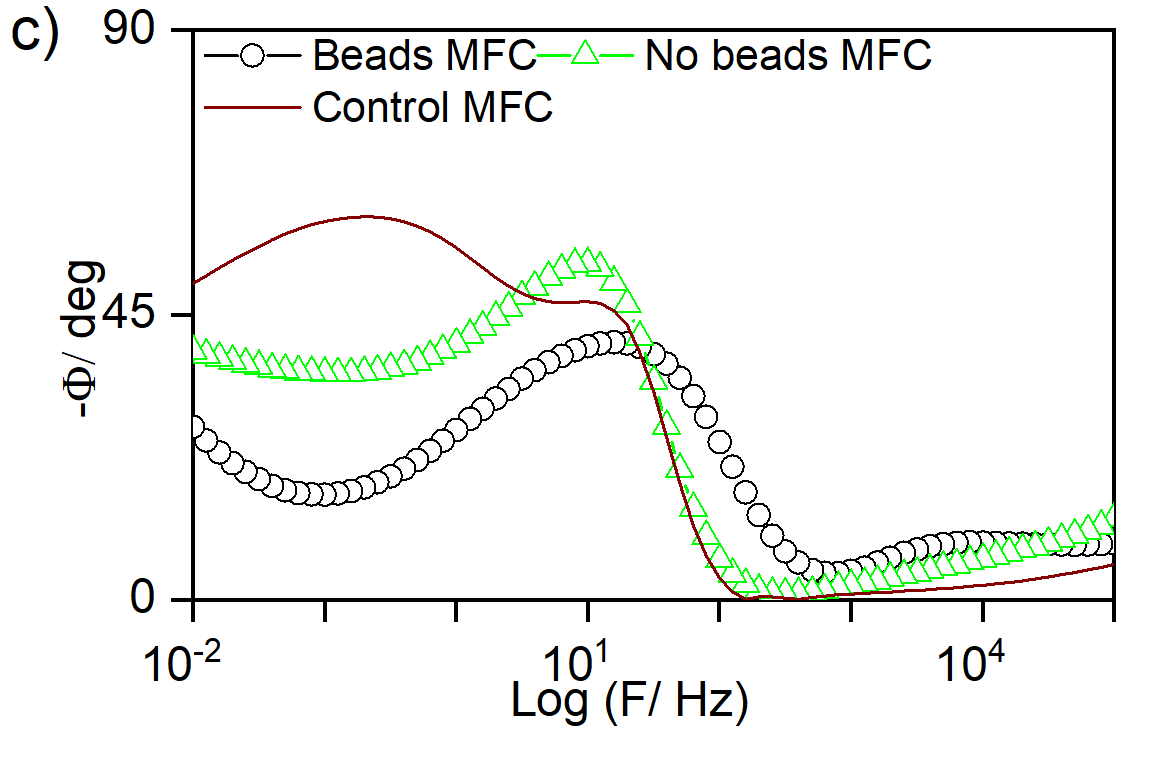


Figure S7 | Electrochemical Impedance spectroscopy analysis of Bead/ No Beads MFC (a) Nyquist plots (b) Modulus plot (c) Phase plots

Table S4. Comparison of rib operon genes in *Bacillus subtilis* and *Geobacillus sp.* WSCUCF1

| *Bacillus subtilis* | Accession No. | *Geobacillus sp. WSUCF1* | Accession No. | EC Number |
| --- | --- | --- | --- | --- |
| 1) Fused 3,4-dihydroxy-2-butanone-4-phosphate synthase  2) GTP Cyclohydrolase II | NP_390207.1  NP_390207.1 | 3,4-dihydroxy-2-butanone-4-phosphate synthase, partial.  GTP Cyclohydrolase II, partial. | WP_033006695.1  WP_033005260.1 | EC 4.1.99.12  EC 3.5.4.25 |
| 3) 6,7-dimethyl-8-ribityllumazine synthase, beta subunit | NP_390206.1 | Multispecies: 6,7-dimethyl-8-ribityllumazine synthase, beta subunit | WP_033005258.1 | EC 2.5.1.78 |
| 4) Riboflavin synthase, alpha subunit | NP_390208.1 | Multispecies: Riboflavin synthase | WP_020756695.1 | EC 2.5.1.9 |

Table S5. List of Uncharacterized proteins

|  |  |  |  | Exclusive Unique Peptide Count | | | |
| --- | --- | --- | --- | --- | --- | --- | --- |
| Identified Proteins (154/1720) | Accession Number | Molecular Weight |  | Cytosolic Fraction | Membrane fraction | | Cellular localization |
| Uncharacterized protein OS=Geobacillus virus E3 GN=E3_0104 PE=4 SV=1 | tr\|A0A0H3UZ77\|A0A0H3UZ77_9CAUD | 38 kDa |  | 2 | - |  | Cytoplasmic |
| Uncharacterized protein OS=Geobacillus thermoleovorans B23 GN=B23_0724 PE=4 SV=1 | tr\|A0A098KZQ0\|A0A098KZQ0_GEOTH | 6 kDa |  | - | 1 |  | Unknown |
| Uncharacterized protein OS=Geobacillus thermoleovorans B23 GN=B23_0461 PE=4 SV=1 | tr\|A0A098KXZ7\|A0A098KXZ7_GEOTH (+3) | 15 kDa |  | - | 3 |  | Unknown |
| Uncharacterized protein OS=Geobacillus thermoglucosidasius GN=GT23_0868 PE=4 SV=1 | tr\|A0A178U3J0\|A0A178U3J0_GEOTM (+1) | 47 kDa |  | - | 6 |  | Cytoplasmic Membrane |
| Uncharacterized protein OS=Geobacillus thermoglucosidasius GN=BAA00_20645 PE=4 SV=1 | tr\|A0A1Y3PRP8\|A0A1Y3PRP8_GEOTM | 42 kDa |  | - | 3 |  | Cytoplasmic |
| Uncharacterized protein OS=Geobacillus thermoglucosidasius GN=BAA00_18505 PE=4 SV=1 | tr\|A0A1Y3PS11\|A0A1Y3PS11_GEOTM | 36 kDa |  | 7 | 10 |  | Cytoplasmic |
| Uncharacterized protein OS=Geobacillus thermoglucosidasius GN=A7K69_11805 PE=4 SV=1 | tr\|A0A1B7KPS1\|A0A1B7KPS1_GEOTM (+3) | 46 kDa |  | 2 | 2 |  | Cytoplasmic Membrane |
| Uncharacterized protein OS=Geobacillus thermoglucosidasius GN=A7K69_07535 PE=4 SV=1 | tr\|A0A1B7KRS0\|A0A1B7KRS0_GEOTM (+3) | 16 kDa |  | - | 6 |  | Outer membrane |
| Uncharacterized protein OS=Geobacillus thermoglucosidasius GN=A7K69_04515 PE=4 SV=1 | tr\|A0A1B7KT52\|A0A1B7KT52_GEOTM | 46 kDa |  | - | 1 |  | Cytoplasmic |
| Uncharacterized protein OS=Geobacillus galactosidasius GN=B9L23_07050 PE=4 SV=1 | tr\|A0A226QQ61\|A0A226QQ61_9BACI (+2) | 46 kDa |  | - | 1 |  | Cytoplasmic |
| Uncharacterized protein OS=Geobacillus thermoglucosidasius GN=A7K69_00790 PE=4 SV=1 | tr\|A0A1B7KW40\|A0A1B7KW40_GEOTM | 22 kDa |  | - | 3 |  | Cytoplasmic |
| Uncharacterized protein OS=Geobacillus thermodenitrificans GN=GTHT12_03309 PE=4 SV=1 | tr\|A0A1W6VMJ2\|A0A1W6VMJ2_GEOTD | 6 kDa |  | 2 |  |  | Cytoplasmic |
| Uncharacterized protein OS=Geobacillus thermocatenulatus GN=GT3921_03080 PE=4 SV=1 | tr\|A0A226QAF1\|A0A226QAF1_9BACI | 36 kDa |  | - | 2 |  | Unknown |
| Uncharacterized protein OS=Geobacillus stearothermophilus GN=pGS18_ORF52 PE=4 SV=1 | tr\|B1GUT3\|B1GUT3_GEOSE | 43 kDa |  | 1 | 10 |  | Cytoplasmic |
| Uncharacterized protein OS=Geobacillus galactosidasius GN=B9L23_19455 PE=4 SV=1 | tr\|A0A226QMN6\|A0A226QMN6_9BACI (+1) | 74 kDa |  | - | 2 |  | Cytoplasmic |
| Uncharacterized protein OS=Geobacillus stearothermophilus GN=B4109_3093 PE=4 SV=1 | tr\|A0A150MT38\|A0A150MT38_GEOSE (+5) | 25 kDa |  | 4 |  |  | Cytoplasmic |
| Uncharacterized protein OS=Geobacillus stearothermophilus GN=B4109_2770 PE=4 SV=1 | tr\|A0A150MJ43\|A0A150MJ43_GEOSE | 50 kDa |  | - | 2 |  | Unknown |
| Uncharacterized protein OS=Geobacillus sp. 1017 GN=BRO54_2093 PE=4 SV=1 | tr\|A0A1Q5SYJ7\|A0A1Q5SYJ7_9BACI | 51 kDa |  | - | 1 |  | Outer membrane |
| Uncharacterized protein OS=Geobacillus stearothermophilus GN=AA904_06605 PE=4 SV=1 | tr\|A0A0K9HSY8\|A0A0K9HSY8_GEOSE (+4) | 59 kDa |  |  | 1 |  | Cytoplasmic Membrane |
| Uncharacterized protein OS=Geobacillus sp. Sah69 GN=AP057_02275 PE=4 SV=1 | tr\|A0A0Q1CQC7\|A0A0Q1CQC7_9BACI | 29 kDa |  | 1 | - |  | Cytoplasmic |
| Uncharacterized protein OS=Geobacillus sp. PA-3 GN=GEPA3_3574 PE=4 SV=1 | tr\|A0A0Q1EEL1\|A0A0Q1EEL1_9BACI | 10 kDa |  | 10 | - |  | Cytoplasmic |
| Uncharacterized protein OS=Geobacillus sp. PA-3 GN=GEPA3_3186 PE=4 SV=1 | tr\|A0A0Q1EG23\|A0A0Q1EG23_9BACI | 4 kDa |  | 1 | - |  | Unknown |
| Uncharacterized protein OS=Geobacillus sp. PA-3 GN=GEPA3_1967 PE=4 SV=1 | tr\|A0A0Q0UUI6\|A0A0Q0UUI6_9BACI (+1) | 21 kDa |  | - | 4 |  | Cytoplasmic |
| Uncharacterized protein OS=Geobacillus sp. LEMMY01 GN=B1A75_18880 PE=4 SV=1 | tr\|A0A1V4P0S7\|A0A1V4P0S7_9BACI | 10 kDa |  | 5 | 1 |  | Cytoplasmic |
| Uncharacterized protein OS=Geobacillus sp. LEMMY01 GN=B1A75_06080 PE=4 SV=1 | tr\|A0A1V4P9Q9\|A0A1V4P9Q9_9BACI | 80 kDa |  | 2 | - |  | Unknown |
| Uncharacterized protein OS=Geobacillus sp. JS12 GN=A0V43_08655 PE=4 SV=1 | tr\|A0A142D1P6\|A0A142D1P6_9BACI (+1) | 9 kDa |  | 2 | 3 |  | Cytoplasmic |
| Uncharacterized protein OS=Geobacillus sp. GHH01 GN=GHH_c21690 PE=4 SV=1 | tr\|L8A000\|REVERSE_L8A000_9BACI-DECOY | 15 kDa |  | 2 | 2 |  | Cytoplasmic |
| Uncharacterized protein OS=Geobacillus sp. CAMR12739 GN=DI43_12375 PE=4 SV=1 | tr\|A0A063YJ14\|A0A063YJ14_9BACI (+13) | 20 kDa |  | - | 1 |  | Unknown |
| Uncharacterized protein OS=Geobacillus sp. CAMR12739 GN=DI43_03170 PE=4 SV=1 | tr\|A0A063YV81\|A0A063YV81_9BACI (+6) | 12 kDa |  | - | 1 |  | Unknown |
| Uncharacterized protein OS=Geobacillus sp. CAMR5420 GN=DI44_16975 PE=4 SV=1 | tr\|A0A063YPZ8\|A0A063YPZ8_9BACI (+5) | 15 kDa |  | - | 1 |  | Unknown |
| Uncharacterized protein OS=Geobacillus sp. CAMR5420 GN=DI44_16860 PE=4 SV=1 | tr\|A0A063YMG6\|A0A063YMG6_9BACI (+24) | 13 kDa |  | - | 2 |  | Cytoplasmic Membrane |
| Uncharacterized protein OS=Geobacillus sp. CAMR5420 GN=DI44_07015 PE=4 SV=1 | tr\|A0A063YXC2\|A0A063YXC2_9BACI (+12) | 28 kDa |  | - | 3 |  | Cytoplasmic |
| Uncharacterized protein OS=Geobacillus sp. B4113_201601 GN=B4113_3771 PE=4 SV=1 | tr\|A0A150MVI3\|A0A150MVI3_9BACI (+1) | 11 kDa |  | - | 8 |  | Cytoplasmic |
| Uncharacterized protein OS=Geobacillus sp. B4113_201601 GN=B4113_0846 PE=4 SV=1 | tr\|A0A150MQ23\|A0A150MQ23_9BACI (+2) | 73 kDa |  | 7 | 17 |  | Cytoplasmic Membrane |
| Uncharacterized protein OS=Geobacillus sp. B4113_201601 GN=B4113_0346 PE=4 SV=1 | tr\|A0A150MRE6\|A0A150MRE6_9BACI | 48 kDa |  | - | 10 |  | Outer membrane |
| Uncharacterized protein OS=Geobacillus sp. AYN2 GN=CN643_17385 PE=4 SV=1 | tr\|A0A2A6AHH2\|A0A2A6AHH2_9BACI | 28 kDa |  | - | 2 |  | Unknown |
| Uncharacterized protein OS=Geobacillus sp. AYN2 GN=CN643_16610 PE=4 SV=1 | tr\|A0A2A6AHE1\|A0A2A6AHE1_9BACI | 22 kDa |  | 2 | - |  | Unknown |
| Uncharacterized protein OS=Geobacillus galactosidasius GN=B9L23_18780 PE=4 SV=1 | tr\|A0A226QMP8\|A0A226QMP8_9BACI | 23 kDa |  | 3 | - |  | Unknown |
| Uncharacterized protein OS=Geobacillus sp. AYN2 GN=CN643_15715 PE=4 SV=1 | tr\|A0A2A6AJ17\|A0A2A6AJ17_9BACI (+1) | 46 kDa |  | - | 5 |  | Unknown |
| Uncharacterized protein OS=Geobacillus thermoleovorans GN=A5N86_08215 PE=3 SV=1 | tr\|A0A1C3D9N2\|A0A1C3D9N2_GEOTH | 40 kDa |  | - | 4 |  | Unknown |
| Uncharacterized protein OS=Geobacillus sp. AYN2 GN=CN643_12720 PE=4 SV=1 | tr\|A0A2A6AQ75\|A0A2A6AQ75_9BACI | 39 kDa |  | 3 | 2 |  | Unknown |
| Uncharacterized protein OS=Geobacillus sp. AYN2 GN=CN643_12530 PE=4 SV=1 | tr\|A0A2A6AP88\|A0A2A6AP88_9BACI (+1) | 32 kDa |  | 1 | - |  | Unknown |
| Uncharacterized protein OS=Geobacillus sp. AYN2 GN=CN643_10910 PE=4 SV=1 | tr\|A0A2A6APB5\|A0A2A6APB5_9BACI (+1) | 37 kDa |  | - | 1 |  | Unknown |
| Uncharacterized protein OS=Geobacillus galactosidasius GN=B9L23_12170 PE=4 SV=1 | tr\|A0A226QKP5\|A0A226QKP5_9BACI | 37 kDa |  | - | 4 |  | Unknown |
| Uncharacterized protein OS=Geobacillus sp. AYN2 GN=CN643_08165 PE=4 SV=1 | tr\|A0A2A6AMG4\|A0A2A6AMG4_9BACI | 26 kDa |  | - | 2 |  | Unknown |
| Uncharacterized protein OS=Geobacillus sp. AYN2 GN=CN643_06905 PE=4 SV=1 | tr\|A0A2A6AKU7\|A0A2A6AKU7_9BACI (+1) | 20 kDa |  | - | 5 |  | Outer membrane |
| Uncharacterized protein OS=Geobacillus sp. AYN2 GN=CN643_00795 PE=4 SV=1 | tr\|A0A2A6AJ43\|A0A2A6AJ43_9BACI | 13 kDa |  | 1 | 2 |  | Cytoplasmic |
| Uncharacterized protein OS=Geobacillus sp. AYN2 GN=CN643_00760 PE=4 SV=1 | tr\|A0A2A6AIL7\|A0A2A6AIL7_9BACI (+1) | 15 kDa |  | - | 2 |  | Cytoplasmic Membrane |
| Uncharacterized protein OS=Geobacillus sp. AYN2 GN=CN643_00520 PE=4 SV=1 | tr\|A0A2A6AIH2\|A0A2A6AIH2_9BACI | 37 kDa |  | - | 3 |  | Cytoplasmic Membrane |
| Uncharacterized protein OS=Geobacillus sp. AYN2 GN=CN643_00485 PE=4 SV=1 | tr\|A0A2A6AI78\|A0A2A6AI78_9BACI | 52 kDa |  | - | 1 |  | Cytoplasmic |
| Uncharacterized protein OS=Geobacillus sp. (strain Y412MC10) GN=GYMC10_0660 PE=4 SV=1 | tr\|D3ECS0\|D3ECS0_GEOS4 | 5 kDa |  | - | 5 |  | Cytoplasmic Membrane |
| Uncharacterized protein OS=Geobacillus sp. (strain Y4.1MC1) GN=GY4MC1_3628 PE=4 SV=1 | tr\|A0A0F6BS63\|A0A0F6BS63_GEOS0 | 20 kDa |  | 4 | 6 |  | Unknown |
| Uncharacterized protein OS=Geobacillus sp. (strain Y4.1MC1) GN=GY4MC1_2726 PE=4 SV=1 | tr\|A0A0F6BPS1\|A0A0F6BPS1_GEOS0 (+2) | 13 kDa |  | 3 | 5 |  | Unknown |
| Uncharacterized protein OS=Geobacillus sp. (strain Y4.1MC1) GN=GY4MC1_1797 PE=4 SV=1 | tr\|A0A0F6BMB8\|A0A0F6BMB8_GEOS0 (+7) | 49 kDa |  | - | 1 |  | Cytoplasmic |
| Uncharacterized protein OS=Geobacillus sp. (strain Y4.1MC1) GN=GY4MC1_1171 PE=4 SV=1 | tr\|A0A0F6BKN1\|A0A0F6BKN1_GEOS0 (+2) | 14 kDa |  | 2 | 2 |  | Cytoplasmic Membrane |
| Uncharacterized protein OS=Geobacillus sp. (strain Y4.1MC1) GN=GY4MC1_0387 PE=4 SV=1 | tr\|A0A0F6BIH8\|A0A0F6BIH8_GEOS0 (+1) | 40 kDa |  | - | 1 |  | Unknown |
| Uncharacterized protein OS=Geobacillus sp. (strain WCH70) GN=GWCH70_3277 PE=4 SV=1 | tr\|C5D964\|C5D964_GEOSW | 26 kDa |  | 4 | 3 |  | Unknown |
| Uncharacterized protein OS=Geobacillus sp. (strain WCH70) GN=GWCH70_3242 PE=4 SV=1 | tr\|C5D934\|C5D934_GEOSW | 16 kDa |  | 2 | 6 |  | Unknown |
| Uncharacterized protein OS=Geobacillus sp. (strain WCH70) GN=GWCH70_3095 PE=4 SV=1 | tr\|C5D8B7\|C5D8B7_GEOSW | 14 kDa |  | 1 | 2 |  | Unknown |
| Uncharacterized protein OS=Geobacillus sp. (strain WCH70) GN=GWCH70_3048 PE=4 SV=1 | tr\|C5D877\|C5D877_GEOSW | 33 kDa |  | - | 7 |  | Cytoplasmic |
| Uncharacterized protein OS=Geobacillus sp. (strain WCH70) GN=GWCH70_2877 PE=4 SV=1 | tr\|C5D719\|C5D719_GEOSW | 24 kDa |  | - | 1 |  | Cytoplasmic |
| Uncharacterized protein OS=Geobacillus sp. (strain WCH70) GN=GWCH70_2613 PE=4 SV=1 | tr\|C5D5N9\|C5D5N9_GEOSW | 29 kDa |  | 3 | 2 |  | Unknown |
| Uncharacterized protein OS=Geobacillus sp. (strain WCH70) GN=GWCH70_2570 PE=4 SV=1 | tr\|C5D5J7\|C5D5J7_GEOSW | 52 kDa |  | - | 2 |  | Unknown |
| Uncharacterized protein OS=Geobacillus sp. (strain WCH70) GN=GWCH70_2393 PE=4 SV=1 | tr\|C5D4P8\|C5D4P8_GEOSW | 29 kDa |  | - | 1 |  | Unknown |
| Uncharacterized protein OS=Geobacillus sp. (strain WCH70) GN=GWCH70_2302 PE=4 SV=1 | tr\|C5D450\|C5D450_GEOSW | 14 kDa |  | 1 | 6 |  | Unknown |
| Uncharacterized protein OS=Geobacillus sp. B4113_201601 GN=B4113_0848 PE=4 SV=1 | tr\|A0A150MQ27\|A0A150MQ27_9BACI | 13 kDa |  | - | 1 |  | Cytoplasmic Membrane |
| Uncharacterized protein OS=Geobacillus sp. (strain WCH70) GN=GWCH70_2299 PE=4 SV=1 | tr\|C5D448\|C5D448_GEOSW | 13 kDa |  | 2 | 2 |  | Cytoplasmic Membrane |
| Uncharacterized protein OS=Geobacillus sp. (strain WCH70) GN=GWCH70_2230 PE=4 SV=1 | tr\|C5D3M3\|C5D3M3_GEOSW | 19 kDa |  | 2 | 6 |  | Outer membrane |
| Uncharacterized protein OS=Geobacillus sp. (strain WCH70) GN=GWCH70_2213 PE=4 SV=1 | tr\|C5D3K7\|C5D3K7_GEOSW | 23 kDa |  | 2 | 7 |  | Unknown |
| Uncharacterized protein OS=Geobacillus sp. (strain WCH70) GN=GWCH70_2210 PE=4 SV=1 | tr\|C5D3K4\|C5D3K4_GEOSW | 55 kDa |  | - | 4 |  | Cytoplasmic Membrane |
| Uncharacterized protein OS=Geobacillus sp. (strain WCH70) GN=GWCH70_2049 PE=4 SV=1 | tr\|C5D2X9\|C5D2X9_GEOSW | 46 kDa |  | - | 2 |  | Cytoplasmic |
| Uncharacterized protein OS=Geobacillus galactosidasius GN=B9L23_12870 PE=4 SV=1 | tr\|A0A226QHP1\|A0A226QHP1_9BACI | 49 kDa |  | - | 1 |  | Unknown |
| Uncharacterized protein OS=Geobacillus sp. (strain WCH70) GN=GWCH70_1898 PE=4 SV=1 | tr\|C5D2J1\|C5D2J1_GEOSW | 6 kDa |  | 2 | 2 |  | Unknown |
| Uncharacterized protein OS=Geobacillus sp. (strain WCH70) GN=GWCH70_1573 PE=3 SV=1 | tr\|C5DAQ6\|C5DAQ6_GEOSW | 16 kDa |  | - | 2 |  | Cell wall |
| Uncharacterized protein OS=Geobacillus sp. (strain WCH70) GN=GWCH70_1324 PE=4 SV=1 | tr\|C5DA55\|C5DA55_GEOSW | 15 kDa |  | 2 | 2 |  | Unknown |
| Uncharacterized protein OS=Geobacillus sp. (strain WCH70) GN=GWCH70_1312 PE=4 SV=1 | tr\|C5DA45\|C5DA45_GEOSW | 23 kDa |  | - | 3 |  | Unknown |
| Uncharacterized protein OS=Geobacillus sp. AYN2 GN=CN643_08300 PE=4 SV=1 | tr\|A0A2A6AM99\|A0A2A6AM99_9BACI | 49 kDa |  | - | 1 |  | Unknown |
| Uncharacterized protein OS=Geobacillus sp. (strain WCH70) GN=GWCH70_1290 PE=4 SV=1 | tr\|C5DA26\|C5DA26_GEOSW | 49 kDa |  | 1 | 4 |  | Unknown |
| Uncharacterized protein OS=Geobacillus sp. (strain WCH70) GN=GWCH70_0991 PE=4 SV=1 | tr\|C5D8J4\|C5D8J4_GEOSW | 14 kDa |  | 5 | 6 |  | Unknown |
| Uncharacterized protein OS=Geobacillus sp. (strain WCH70) GN=GWCH70_0971 PE=4 SV=1 | tr\|C5D852\|C5D852_GEOSW | 11 kDa |  | - | 1 |  | Unknown |
| Uncharacterized protein OS=Geobacillus sp. (strain WCH70) GN=GWCH70_0934 PE=4 SV=1 | tr\|C5D816\|C5D816_GEOSW | 20 kDa |  | - | 1 |  | Unknown |
| Uncharacterized protein OS=Geobacillus sp. (strain WCH70) GN=GWCH70_0890 PE=4 SV=1 | tr\|C5D7X8\|C5D7X8_GEOSW | 27 kDa |  | 22 | 31 |  | Unknown |
| Uncharacterized protein OS=Geobacillus sp. 44B GN=BSK33_16850 PE=4 SV=1 | tr\|A0A1V9ANS7\|A0A1V9ANS7_9BACI | 27 kDa |  | 11 | - |  | Unknown |
| Uncharacterized protein OS=Geobacillus sp. (strain WCH70) GN=GWCH70_0873 PE=4 SV=1 | tr\|C5D7W5\|C5D7W5_GEOSW | 28 kDa |  | - | 4 |  | Unknown |
| Uncharacterized protein OS=Geobacillus sp. (strain WCH70) GN=GWCH70_0730 PE=3 SV=1 | tr\|C5D758\|C5D758_GEOSW | 28 kDa |  | 1 | 1 |  | Unknown |
| Uncharacterized protein OS=Geobacillus sp. (strain WCH70) GN=GWCH70_0657 PE=4 SV=1 | tr\|C5D6N3\|C5D6N3_GEOSW | 36 kDa |  | 3 | 3 |  | Cytoplasmic |
| Uncharacterized protein OS=Geobacillus sp. (strain WCH70) GN=GWCH70_0582 PE=4 SV=1 | tr\|C5D6G5\|C5D6G5_GEOSW | 14 kDa |  | 4 | 7 |  | Cytoplasmic |
| Uncharacterized protein OS=Geobacillus sp. (strain WCH70) GN=GWCH70_0521 PE=4 SV=1 | tr\|C5D5Y2\|C5D5Y2_GEOSW | 51 kDa |  | - | 2 |  | Unknown |
| Uncharacterized protein OS=Geobacillus sp. (strain WCH70) GN=GWCH70_0403 PE=4 SV=1 | tr\|C5D597\|C5D597_GEOSW | 13 kDa |  | - | 1 |  | Cytoplasmic |
| Uncharacterized protein OS=Geobacillus sp. (strain WCH70) GN=GWCH70_0329 PE=4 SV=1 | tr\|C5D530\|C5D530_GEOSW | 23 kDa |  | - | 2 |  | Unknown |
| Uncharacterized protein OS=Geobacillus sp. (strain WCH70) GN=GWCH70_0301 PE=4 SV=1 | tr\|C5D4M0\|C5D4M0_GEOSW | 47 kDa |  | 1 | 4 |  | Unknown |
| Uncharacterized protein OS=Geobacillus sp. (strain WCH70) GN=GWCH70_0285 PE=4 SV=1 | tr\|C5D4K4\|C5D4K4_GEOSW | 23 kDa |  | 2 | - |  | Cytoplasmic |
| Uncharacterized protein OS=Geobacillus sp. (strain WCH70) GN=GWCH70_0216 PE=4 SV=1 | tr\|C5D4D9\|C5D4D9_GEOSW | 17 kDa |  | - | 2 |  | Cytoplasmic Membrane |
| Uncharacterized protein OS=Geobacillus sp. (strain WCH70) GN=GWCH70_0174 PE=4 SV=1 | tr\|C5D3X5\|C5D3X5_GEOSW | 24 kDa |  | - | 4 |  | Cytoplasmic Membrane |
| Uncharacterized protein OS=Geobacillus sp. (strain WCH70) GN=GWCH70_0060 PE=4 SV=1 | tr\|C5D386\|C5D386_GEOSW | 27 kDa |  | - | 3 |  | Cytoplasmic Membrane |
| Uncharacterized protein OS=Geobacillus sp. 44B GN=BSK33_13100 PE=4 SV=1 | tr\|A0A1V9AUJ2\|A0A1V9AUJ2_9BACI | 94 kDa |  | 1 | 1 |  | Unknown |
| Uncharacterized protein OS=Geobacillus sp. 44B GN=BSK33_07335 PE=4 SV=1 | tr\|A0A1V9B1U1\|A0A1V9B1U1_9BACI | 15 kDa |  | - | 2 |  | Unknown |
| Uncharacterized protein OS=Geobacillus sp. 44B GN=BSK33_07235 PE=4 SV=1 | tr\|A0A1V9B202\|A0A1V9B202_9BACI (+1) | 9 kDa |  | - | 2 |  | Unknown |
| Uncharacterized protein OS=Geobacillus sp. 44B GN=BSK33_03990 PE=4 SV=1 | tr\|A0A1V9B4D1\|A0A1V9B4D1_9BACI (+3) | 23 kDa |  | - | 3 |  | Unknown |
| Uncharacterized protein OS=Geobacillus sp. 44B GN=BSK33_00775 PE=3 SV=1 | tr\|A0A1V9B7T9\|A0A1V9B7T9_9BACI (+1) | 16 kDa |  | 2 | - |  | Cytoplasmic |
| Uncharacterized protein OS=Geobacillus sp. 15 GN=A3Q36_15995 PE=4 SV=1 | tr\|A0A164C6R8\|A0A164C6R8_9BACI | 20 kDa |  | - | 1 |  | Cytoplasmic |
| Uncharacterized protein OS=Geobacillus sp. 15 GN=A3Q36_10315 PE=4 SV=1 | tr\|A0A163YEN9\|A0A163YEN9_9BACI | 37 kDa |  | 11 | - |  | Unknown |
| Uncharacterized protein OS=Geobacillus sp. (strain WCH70) GN=GWCH70_1715 PE=4 SV=1 | tr\|C5D237\|C5D237_GEOSW | 15 kDa |  | 1 | 8 |  | Unknown |
| Uncharacterized protein OS=Geobacillus lituanicus GN=GLN3_15395 PE=4 SV=1 | tr\|A0A223DZ76\|A0A223DZ76_9BACI | 15 kDa |  | - | 2 |  | Unknown |
| Uncharacterized protein OS=Geobacillus kaustophilus GBlys GN=GBL_2469 PE=4 SV=1 | tr\|U2X661\|U2X661_GEOKU | 29 kDa |  | 3 | 1 |  | Cytoplasmic |
| Uncharacterized protein OS=Geobacillus galactosidasius GN=B9L23_19505 PE=4 SV=1 | tr\|A0A226QP64\|A0A226QP64_9BACI | 25 kDa |  | 1 | - |  | Cytoplasmic Membrane |
| Uncharacterized protein OS=Geobacillus galactosidasius GN=B9L23_18315 PE=4 SV=1 | tr\|A0A226QNP2\|A0A226QNP2_9BACI (+2) | 49 kDa |  | - | 1 |  | Cytoplasmic Membrane |
| Uncharacterized protein OS=Geobacillus galactosidasius GN=B9L23_17945 PE=4 SV=1 | tr\|A0A226QKC6\|A0A226QKC6_9BACI (+1) | 46 kDa |  | - | 2 |  | Cytoplasmic |
| Uncharacterized protein OS=Geobacillus galactosidasius GN=B9L23_17070 PE=4 SV=1 | tr\|A0A226QJW6\|A0A226QJW6_9BACI | 17 kDa |  | - | 3 |  | Cytoplasmic |
| Uncharacterized protein OS=Geobacillus galactosidasius GN=B9L23_16480 PE=4 SV=1 | tr\|A0A226QM35\|A0A226QM35_9BACI (+1) | 37 kDa |  | 2 | 3 |  | Cytoplasmic |
| Uncharacterized protein OS=Geobacillus galactosidasius GN=B9L23_15770 PE=4 SV=1 | tr\|A0A226QLA7\|A0A226QLA7_9BACI (+1) | 44 kDa |  | - | 1 |  | Unknown |
| Uncharacterized protein OS=Geobacillus galactosidasius GN=B9L23_15435 PE=4 SV=1 | tr\|A0A226QLG6\|A0A226QLG6_9BACI (+2) | 55 kDa |  | 3 | 1 |  | Unknown |
| Uncharacterized protein OS=Geobacillus galactosidasius GN=B9L23_15095 PE=4 SV=1 | tr\|A0A226QLF8\|A0A226QLF8_9BACI | 25 kDa |  | 9 | 1 |  | Unknown |
| Uncharacterized protein OS=Geobacillus galactosidasius GN=B9L23_15010 PE=4 SV=1 | tr\|A0A226QLP6\|A0A226QLP6_9BACI (+1) | 24 kDa |  | 1 |  |  | Unknown |
| Uncharacterized protein OS=Geobacillus galactosidasius GN=B9L23_14925 PE=4 SV=1 | tr\|A0A226QJ99\|A0A226QJ99_9BACI (+1) | 20 kDa |  | - | 2 |  | Cytoplasmic |
| Uncharacterized protein OS=Geobacillus galactosidasius GN=B9L23_14775 PE=4 SV=1 | tr\|A0A226QM20\|A0A226QM20_9BACI | 17 kDa |  | 3 |  |  | Unknown |
| Uncharacterized protein OS=Geobacillus galactosidasius GN=B9L23_14370 PE=4 SV=1 | tr\|A0A226QIG8\|A0A226QIG8_9BACI | 35 kDa |  | - | 3 |  | Cytoplasmic |
| Uncharacterized protein OS=Geobacillus galactosidasius GN=B9L23_13565 PE=4 SV=1 | tr\|A0A226QI12\|A0A226QI12_9BACI (+1) | 37 kDa |  | - | 4 |  | Cytoplasmic Membrane |
| Uncharacterized protein OS=Geobacillus galactosidasius GN=B9L23_13445 PE=4 SV=1 | tr\|A0A226QHY5\|A0A226QHY5_9BACI (+1) | 28 kDa |  | 1 | 1 |  | Cytoplasmic |
| Uncharacterized protein OS=Geobacillus galactosidasius GN=B9L23_13330 PE=4 SV=1 | tr\|A0A226QL72\|A0A226QL72_9BACI (+1) | 49 kDa |  | - | 3 |  | Unknown |
| Uncharacterized protein OS=Geobacillus galactosidasius GN=B9L23_13320 PE=4 SV=1 | tr\|A0A226QKS8\|A0A226QKS8_9BACI (+1) | 18 kDa |  | - | 1 |  | Unknown |
| Uncharacterized protein OS=Geobacillus galactosidasius GN=B9L23_12035 PE=4 SV=1 | tr\|A0A226QJD0\|A0A226QJD0_9BACI (+1) | 29 kDa |  | - | 1 |  | Unknown |
| Uncharacterized protein OS=Geobacillus galactosidasius GN=B9L23_11750 PE=4 SV=1 | tr\|A0A226QK42\|A0A226QK42_9BACI | 8 kDa |  | - | 3 |  | Unknown |
| Uncharacterized protein OS=Geobacillus galactosidasius GN=B9L23_11435 PE=4 SV=1 | tr\|A0A226QKJ9\|A0A226QKJ9_9BACI | 7 kDa |  | 3 | 6 |  | Unknown |
| Uncharacterized protein OS=Geobacillus galactosidasius GN=B9L23_11045 PE=4 SV=1 | tr\|A0A226QJH6\|A0A226QJH6_9BACI (+1) | 36 kDa |  | - | 2 |  | Unknown |
| Uncharacterized protein OS=Geobacillus galactosidasius GN=B9L23_10475 PE=4 SV=1 | tr\|A0A226QIP3\|A0A226QIP3_9BACI | 72 kDa |  | 1 | 1 |  | Unknown |
| Uncharacterized protein OS=Geobacillus galactosidasius GN=B9L23_10375 PE=4 SV=1 | tr\|A0A226QJW8\|A0A226QJW8_9BACI (+1) | 29 kDa |  | - | 1 |  | Unknown |
| Uncharacterized protein OS=Geobacillus galactosidasius GN=B9L23_10120 PE=4 SV=1 | tr\|A0A226QJT5\|A0A226QJT5_9BACI | 20 kDa |  | 1 | 2 |  | Unknown |
| Uncharacterized protein OS=Geobacillus galactosidasius GN=B9L23_09855 PE=4 SV=1 | tr\|A0A226QGD2\|A0A226QGD2_9BACI | 163 kDa |  | - | 1 |  | Unknown |
| Uncharacterized protein OS=Geobacillus galactosidasius GN=B9L23_09575 PE=3 SV=1 | tr\|A0A226QG24\|A0A226QG24_9BACI (+2) | 20 kDa |  | 1 |  |  | Cytoplasmic |
| Uncharacterized protein OS=Geobacillus galactosidasius GN=B9L23_07115 PE=4 SV=1 | tr\|A0A226QQ40\|A0A226QQ40_9BACI | 33 kDa |  | 2 | 2 |  | Cytoplasmic Membrane |
| Uncharacterized protein OS=Geobacillus sp. 8 GN=A3Q35_00635 PE=4 SV=1 | tr\|A0A164C0D2\|A0A164C0D2_9BACI | 61 kDa |  | - | 2 |  | Cell wall |
| Uncharacterized protein OS=Geobacillus galactosidasius GN=B9L23_06215 PE=4 SV=1 | tr\|A0A226QPR2\|A0A226QPR2_9BACI | 61 kDa |  | - | 7 |  | Outer membrane |
| Uncharacterized protein OS=Geobacillus galactosidasius GN=B9L23_05875 PE=4 SV=1 | tr\|A0A226QPG7\|A0A226QPG7_9BACI (+1) | 21 kDa |  | - | 3 |  | Cytoplasmic |
| Uncharacterized protein OS=Geobacillus galactosidasius GN=B9L23_05720 PE=4 SV=1 | tr\|A0A226QRN9\|A0A226QRN9_9BACI | 17 kDa |  | 4 | 2 |  | Cytoplasmic |
| Uncharacterized protein OS=Geobacillus galactosidasius GN=B9L23_05570 PE=4 SV=1 | tr\|A0A226QS58\|A0A226QS58_9BACI | 18 kDa |  | - | 2 |  | Cytoplasmic |
| Uncharacterized protein OS=Geobacillus galactosidasius GN=B9L23_04285 PE=4 SV=1 | tr\|A0A226QRT8\|A0A226QRT8_9BACI (+1) | 32 kDa |  | - | 3 |  | Cytoplasmic |
| Uncharacterized protein OS=Geobacillus galactosidasius GN=B9L23_04050 PE=4 SV=1 | tr\|A0A226QRE8\|A0A226QRE8_9BACI | 34 kDa |  | - | 6 |  | Unknown |
| Uncharacterized protein OS=Geobacillus thermoglucosidasius GN=GT23_3269 PE=4 SV=1 | tr\|A0A178TTT9\|A0A178TTT9_GEOTM (+1) | 28 kDa |  | 4 | 1 |  | Cytoplasmic |
| Uncharacterized protein OS=Geobacillus galactosidasius GN=B9L23_03100 PE=4 SV=1 | tr\|A0A226QRA0\|A0A226QRA0_9BACI | 30 kDa |  | 9 | 11 |  | Unknown |
| Uncharacterized protein OS=Geobacillus galactosidasius GN=B9L23_03065 PE=4 SV=1 | tr\|A0A226QQX4\|A0A226QQX4_9BACI (+1) | 25 kDa |  | 8 | 3 |  | Unknown |
| Uncharacterized protein OS=Geobacillus galactosidasius GN=B9L23_02195 PE=4 SV=1 | tr\|A0A226QQL5\|A0A226QQL5_9BACI | 34 kDa |  | 1 | 7 |  | Cytoplasmic Membrane |
| Uncharacterized protein OS=Geobacillus galactosidasius GN=B9L23_02190 PE=4 SV=1 | tr\|A0A226QMN4\|A0A226QMN4_9BACI (+1) | 41 kDa |  | 3 | 5 |  | Outer membrane |
| Uncharacterized protein OS=Geobacillus galactosidasius GN=B9L23_02150 PE=4 SV=1 | tr\|A0A226QN49\|A0A226QN49_9BACI (+2) | 20 kDa |  | - | 2 |  | Unknown |
| Uncharacterized protein OS=Geobacillus galactosidasius GN=B9L23_01785 PE=4 SV=1 | tr\|A0A226QMF9\|A0A226QMF9_9BACI | 13 kDa |  | 10 | 1 |  | Unknown |
| Uncharacterized protein OS=Geobacillus galactosidasius GN=B9L23_01215 PE=4 SV=1 | tr\|A0A226QQH8\|A0A226QQH8_9BACI | 18 kDa |  | 2 | 7 |  | Unknown |
| Uncharacterized protein OS=Geobacillus sp. AYN2 GN=CN643_11680 PE=4 SV=1 | tr\|A0A2A6AP62\|A0A2A6AP62_9BACI | 61 kDa |  | - | 16 |  | Cell wall |
| Uncharacterized protein OS=Geobacillus sp. B4113_201601 GN=B4113_3208 PE=4 SV=1 | tr\|A0A150MHD8\|A0A150MHD8_9BACI | 60 kDa |  | 1 | - |  | Unknown |
| Uncharacterized protein OS=Geobacillus sp. B4113_201601 GN=B4113_2139 PE=4 SV=1 | tr\|A0A150MXX9\|A0A150MXX9_9BACI | 66 kDa |  | 6 | - |  | Cytoplasmic |
| Uncharacterized protein OS=Geobacillus sp. 8 GN=A3Q35_15665 PE=4 SV=1 | tr\|A0A163YSD9\|A0A163YSD9_9BACI | 76 kDa |  | 1 | - |  | Cytoplasmic |
| Uncharacterized protein OS=Geobacillus stearothermophilus GN=TGS27_1641 PE=4 SV=1 | tr\|A0A178TIG3\|A0A178TIG3_GEOSE | 27 kDa |  | - | 3 |  | Unknown |
| Uncharacterized protein OS=Geobacillus galactosidasius GN=B9L23_06115 PE=4 SV=1 | tr\|A0A226QPN9\|A0A226QPN9_9BACI (+1) | 65 kDa |  | - | 12 |  | Cytoplasmic Membrane |
| Uncharacterized protein OS=Geobacillus sp. (strain WCH70) GN=GWCH70_2989 PE=4 SV=1 | tr\|C5D7Q2\|C5D7Q2_GEOSW | 40 kDa |  | 1 | 3 |  | Unknown |
| Uncharacterized protein OS=Geobacillus sp. (strain WCH70) GN=GWCH70_0313 PE=4 SV=1 | tr\|C5D4N2\|C5D4N2_GEOSW | 47 kDa |  | - | 11 |  | Unknown |

Table S6. EET proteins identified from the WSUCF1 genome sequence

| Protein | Accession No. | Subcellular location | Function^a^ |
| --- | --- | --- | --- |
| Membrane-attached Cytochrome c550 | [ATCO01000016.1](https://www.ncbi.nlm.nih.gov/nuccore/ATCO01000016.1) | Cytoplasmic membrane | Heme binding; electron transfer activity^10^ |
| Cytochrome c551 | [ATCO01000030.1](https://www.ncbi.nlm.nih.gov/nuccore/ATCO01000030.1) | Cytoplasmic membrane | Heme protein involved in dissimilative denitrification as a physiological electron donor of nitrite reductase^11^ |
| Type IV pilin | [ATCO01000195.1](https://www.ncbi.nlm.nih.gov/nuccore/ATCO01000195.1) | Inner and outer cell membrane | Biofilm formation and electron transfer^12^ |
| Pili assembly protein pilM | [ATCO01000195.1](https://www.ncbi.nlm.nih.gov/nuccore/ATCO01000195.1) | Inner membrane | Pilus biogenesis and regulation of biofilm formation^13, 14^ |
| Riboflavin transporter  Outer membrane lipoprotein carrier protein | [ATCO01000124.1](https://www.ncbi.nlm.nih.gov/nuccore/ATCO01000124.1)  [ATCO01000007.1](https://www.ncbi.nlm.nih.gov/nuccore/NZ_ATCO01000007.1) | Cytoplasmic membrane  Outer membrane | Extracellular riboflavin secretion^15^  Chaperone mediated electron transport across periplasmic space^16^ |

a – Functions of proteins were taken from the uniport database

**Supplementary References**

1. Bhalla, A., Bischoff, K. M. & Sani, R. K. Highly Thermostable Xylanase Production from A Thermophilic Geobacillus sp. Strain WSUCF1 Utilizing Lignocellulosic Biomass. *Front. Bioeng. Biotech.* **3**, (2015).

2. Shrestha, N.*, et al.* Electricity generation from defective tomatoes. *Bioelectrochemistry* **112**, 67-76 (2016).

3. Shrestha, N.*, et al.* Integrated membrane and microbial fuel cell technologies for enabling energy-efficient effluent Re-use in power plants. *Water Res.* **117**, 37-48 (2017).

4. Shrestha, N., Chilkoor, G., Wilder, J., Ren, Z. J. & Gadhamshetty, V. Comparative performances of microbial capacitive deionization cell and microbial fuel cell fed with produced water from the Bakken shale. *Bioelectrochemistry* **121**, 56-64 (2018).

5. Fapetu, S., Keshavarz, T., Clements, M. & Kyazze, G. Contribution of direct electron transfer mechanisms to overall electron transfer in microbial fuel cells utilising Shewanella oneidensis as biocatalyst. *Biotechnol. Lett.* **38**, 1465-1473 (2016).

6. Sonawane, J. M., Marsili, E. & Ghosh, P. C. Treatment of domestic and distillery wastewater in high surface microbial fuel cells. *Int. J. Hydrogen Energy* **39**, 21819-21827 (2014).

7. Wang, G.*, et al.* Enhancement of riboflavin production by deregulating gluconeogenesis in Bacillus subtilis. *World J. Microbiol. Biotechnol.* **30**, 1893-1900 (2014).

8. Chignell, J. F., De Long, S. K. & Reardon, K. F. Meta-proteomic analysis of protein expression distinctive to electricity-generating biofilm communities in air-cathode microbial fuel cells. *Biotechnol. Biofuels* **11**, 121 (2018).

9. Gundry, R. L.*, et al.* Preparation of proteins and peptides for mass spectrometry analysis in a bottom‐up proteomics workflow. *Curr. Protoc. Mol. Biol.* **90**, 10.25. 11-10.25. 23 (2010).

10. Otten, M. F.*, et al.* Cytochromes c(550), c(552), and c(1) in the electron transport network of Paracoccus denitrificans: redundant or subtly different in function? *J. Bacteriol.* **183**, 7017-7026 (2001).

11. Cutruzzola, F.*, et al.* Pseudomonas aeruginosa cytochrome C(551): probing the role of the hydrophobic patch in electron transfer. *J. Inorg. Biochem.* **88**, 353-361 (2002).

12. Giltner, C. L., Nguyen, Y. & Burrows, L. L. Type IV Pilin Proteins: Versatile Molecular Modules. *Microbiol. Mol. Biol. Rev.* **76**, 740 (2012).

13. Piepenbrink, K. H. & Sundberg, E. J. Motility and adhesion through type IV pili in Gram-positive bacteria. *Biochem. Soc. Trans.* **44**, 1659-1666 (2016).

14. Karuppiah, V. & Derrick, J. P. Structure of the PilM-PilN Inner Membrane Type IV Pilus Biogenesis Complex from Thermus thermophilus. *J. Biol. Chem.* **286**, 24434-24442 (2011).

15. Gutiérrez-Preciado, A.*, et al.* Extensive Identification of Bacterial Riboflavin Transporters and Their Distribution across Bacterial Species. *PloS one* **10**, e0126124 (2015).

16. Light, S. H.*, et al.* A flavin-based extracellular electron transfer mechanism in diverse Gram-positive bacteria. *Nature* **562**, 140-144 (2018).
